# Preclinical studies of Flonoltinib Maleate, a novel JAK2/FLT3 inhibitor, in treatment of *JAK2*^V617F^-induced myeloproliferative neoplasms

**DOI:** 10.1038/s41408-022-00628-2

**Published:** 2022-03-07

**Authors:** Mengshi Hu, Tao Yang, Linyu Yang, Lu Niu, Jinbing Zhu, Ailin Zhao, Mingsong Shi, Xue Yuan, Minghai Tang, Jianhong Yang, Heying Pei, Zhuang Yang, Qiang Chen, Haoyu Ye, Ting Niu, Lijuan Chen

**Affiliations:** 1 State Key Laboratory of Biotherapy and Cancer Center, National Clinical Research Center for Geriatrics, West China Hospital of Sichuan University, Chengdu, China; 2 Department of Hematology and Research Laboratory of Hematology, West China Hospital of Sichuan University, Chengdu, China; 3 Chengdu Zenitar Biomedical Technology Co., Ltd, Chengdu, China

**Keywords:** Myeloproliferative disease, Targeted therapies

## Abstract

Janus kinase 2 (JAK2) hyperactivation by *JAK2*^V617F^ mutation leads to myeloproliferative neoplasms (MPNs) and targeting JAK2 could serve as a promising therapeutic strategy for MPNs. Here, we report that Flonoltinib Maleate (FM), a selective JAK2/FLT3 inhibitor, shows high selectivity for JAK2 over the JAK family. Surface plasmon resonance assays verified that FM had a stronger affinity for the pseudokinase domain JH2 than JH1 of JAK2 and had an inhibitory effect on JAK2 JH2V617F. The cocrystal structure confirmed that FM could stably bind to JAK2 JH2, and FM suppressed endogenous colony formation of primary erythroid progenitor cells from patients with MPNs. In several *JAK2*^V617F^-induced MPN murine models, FM could dose-dependently reduce hepatosplenomegaly and prolong survival. Similar results were observed in *JAK2*^V617F^ bone marrow transplantation mice. FM exhibited strong inhibitory effects on fibrosis of the spleen and bone marrow. Long-term FM treatment showed good pharmacokinetic/pharmacodynamic characteristics with high drug exposure in tumor-bearing tissues and low toxicity. Currently, FM has been approved by the National Medical Products Administration of China (CXHL2000628), and this study will guide clinical trials for patients with MPNs.

## Introduction

Myeloproliferative neoplasms (MPNs) are a family of clonal disorders of hematopoietic stem cells featuring a continuous proliferation of one or more lineage cells in the bone marrow (BM) [1, 2]. Mutations of Janus kinase 2 (*JAK2*), predominantly *JAK2*^V617F^, are discovered in ~95% of patients with polycythemia vera and 50–60% of patients with essential thrombocythemia, as well as primary myelofibrosis (MF) [3–6]. These previous results suggest that JAK2 is an important therapeutic target in the treatment of *JAK2*^V617F^-induced MPNs.

Ruxolitinib, a JAK1/JAK2 inhibitor, is the first drug approved for the treatment of intermediate-2 and high-risk patients with MF and alleviates the splenomegaly and systemic symptoms in patients with MF [7–11]. Despite these clinical benefits, ruxolitinib could cause severe anemia, thrombocytopenia, and granulocyte deficiency, apart from non-specific systemic symptoms, such as diarrhea and fatigue [12–15]. These side effects may be related to the inhibition of JAK1 [16, 17], which is associated with IFN-γ resistance and cancer evasion [18]. Fedratinib, a dual JAK2/FLT3 inhibitor approved in 2019 by the FDA for the treatment of MF, improved the selectivity of JAK2 over the JAK family and simultaneously inhibited FLT3. Fedratinib significantly inhibited disease progression compared to the best available therapies after long-term treatment [19, 20]. However, fedratinib is associated with vitamin B1 deficiency-related Wernicke’s encephalopathy [21, 22]. Therefore, the development of highly selective JAK2/FLT3 inhibitors with reduced toxicities to treat *JAK2*^V617F^-induced MPNs is clinically urgently needed.

JAK2 protein contains seven homology domains (JH1-7), of which JH1 (residues 836-1132) is the kinase domain, and JH2 (residues 543-824) is the pseudokinase domain [23]. JH1 could activate JAK2 through auto-phosphorylation and block the catalytic activity of kinases by blocking ATP and downstream phosphorylation pathway signal transduction [24, 25]. The JH2 domain has no catalytic activity but could negatively regulate the activity of JH1 and inhibit the kinase activity of JAK2 [26]. The V617F mutation site is located above the N-terminus of the JH2 domain [27], and when the valine at position 617 is replaced by phenylalanine with a higher molecular weight, the prolongation of helix C and the interaction between F617 and the helix C phenylalanines 594 and 595 of JH2 domain is stable and activates the JH1 domain [28–31]. Ruxolitinib and fedratinib are the most representative JAK kinase inhibitors for the treatment of MF, and both of them bind to the JAK2 JH1 domain [32, 33]. Recently, Bristol Myers Squibb Company (BMS) has developed a highly selective TYK2 inhibitor, named BMS-986165, which acts by binding to the TYK2 JH2 domain. BMS-986165 has been proven to greatly improve selectivity and reduce side effects [34]. Therefore, the development of highly selective JAK2 inhibitors binding to the JAK2 JH2 (pseudokinase domain) may be of great significance in improving the selectivity for JAK2 over the JAK family for the treatment of *JAK2*^V617F^-driven patients.

Here, we reported that a highly selective JAK2 inhibitor, named Flonoltinib Maleate (FM), had an inhibitory effect on JH1, JH2, and JH2V617F of JAK2, and its crystal structure confirmed that FM could stably bind to the JAK2 JH2 domain, which may contribute to the selectivity for the JAK family. Furthermore, FM showed potent efficacy in cell lines as well as murine models of MPNs, additional to primary cells acquired from patients with MPNs. In various MPN models, FM could significantly reduce tumor burden, suppress disease progression, and extend the survival of mice. In addition, FM exhibited higher drug exposure in the tumor-bearing tissue than the plasma and showed good pharmacokinetic/pharmacodynamic (PK/PD) characteristics and low toxicity. Currently, FM has been approved by the National Medical Productions Administration of China (CXHL2000628), and this research will provide evidence for FM clinical trials in JAK2-driven MPN treatment.

## Materials and methods

### Compounds and reagents

2-((1-(2-fluoro-4-((4-(1-isopropyl-1H-pyrazol-4-yl)-5-methylpyrimidin-2-yl)amino)phenyl)piperidin-4-yl)(methyl)amino)ethan-1-ol (FM) was synthesized in the laboratory. Ruxolitinib phosphate (cat#1092939-17-7) and Fedratinib/TG101348 (cat#936091-26-8) were purchased from Sichuan Shuyan Pharmaceutical Technology Co., Ltd. The dissolving methods of in vitro as well as in vivo experiments are presented in Supplementary Information (SI).

### Cell culture

All the cells have been identified by short tandem repeats profiling after 2017. MV-4-11, Molm-13, Ba/F3-*JAK2*^WT^, and HEL cells (ATCC) were cultured in RPMI 1640 (Cat. No. SH30809.01, Hyclone) supplemented with 10% fetal bovine serum (Cat. No.10099-141, Gibco), and penicillin/streptomycin (Cat. No. SV30010, Hyclone). Ba/F3-*JAK2*^WT^ cell culture medium was supplemented with 10 µg/mL IL-3 (Cat. No. 213-13, PeproTech) and 2-hydroxy-1-ethanethiol (Cat. No. 60-24-2, Sigma). Ba/F3-*JAK2*^V617F^ and Ba/F3-*EPOR*-*JAK2*^V617F^ cells were established via retroviral transduction of the vector MSCV-IRES-GFP carrying the *JAK2*^V617F^ and *EPOR* cDNA with polybrene (Cat. No. H8761, Solarbio). The cells were incubated at 37 °C with 5% CO_2_.

### MTT assay for cell proliferation

For in vitro inhibition assays, cells were seeded in 96-well culture plates with a density of 15,000–20,000 cells/well after the cells reached 70–90% confluence. After overnight incubation, the cells received a supplement of serial concentrations of FM and underwent 72 h incubation at 37 °C under 5% CO_2_. MTT (3-(4, 5-dimethylthiazol-2-yl)-2, 5-diphenyltetrazolium bromide) (Cat. No. 1334GR001; BioFroxx) assay was conducted in order to examine viability. The IC_50_ values were fitted using GraphPad Prism 7.0.

### Colony formation assays

Peripheral blood (PB) or BM specimens of patients with MPNs were acquired from West China Hospital with approval of the West China Hospital of Sichuan University (Chengdu, China) clinical ethics committee. We have obtained a statement confirming that informed consent was obtained from all subjects (clinical information is presented in Table S1). A total of 2 × 10^5^ cells were seeded in a methylcellulose semi-solid medium with an increasing concentration of FM. Colonies were detected at 37 °C, 5% CO_2_, and ≥95% humidity after 14 days.

### In vivo efficacy studies

All animal experiments were approved by the Institutional Animal Care and Use Committee of the Sichuan University. Female NOD/SCID mice and BALB/c nude mice were purchased from the Beijing HFK Bioscience Company. Male BALB/c mice were bought from GemPharmatech Co., Ltd. The animal experiments were not blinded to the groups. Mice were six to eight weeks old upon tumor implantation.

We established three *JAK2*^V617^-induced splenomegaly models. The first was the Ba/F3-*JAK2*^V617F^ disease model. We constructed the V617F mutant *JAK2* gene using molecular biology techniques and transferred the gene to Ba/F3, and then 3 × 10^6^ Ba/F3-*JAK2*^V617F^ cells were injected into BALB/c nude mice through the tail vein to induce splenomegaly and simulate the symptoms of *JAK2*^V617F^ gene mutation-bearing patients. Next, we established the Ba/F3-*EPOR*-*JAK2*^V617F^ disease model using the same modeling method as that of the Ba/F3-*JAK2*^V617F^ disease model. This model induced a greater extent of disease burden, faster onset, and more severe hepatosplenomegaly in mice because both *JAK2*^V617F^ and *EPOR* were transduced in Ba/F3 cells. Finally, we established a *JAK2*^V617F^ bone marrow transplantation (BMT) model based on irradiated mice. As the disease progressed, *JAK2*^V617F^ induced primary splenomegaly, splenic fibrosis, and BM fibrosis. The details of the models and methods are described in SI.

## Results

### Potent and highly selective inhibition of JAK2 by FM

**Fig. 1:**
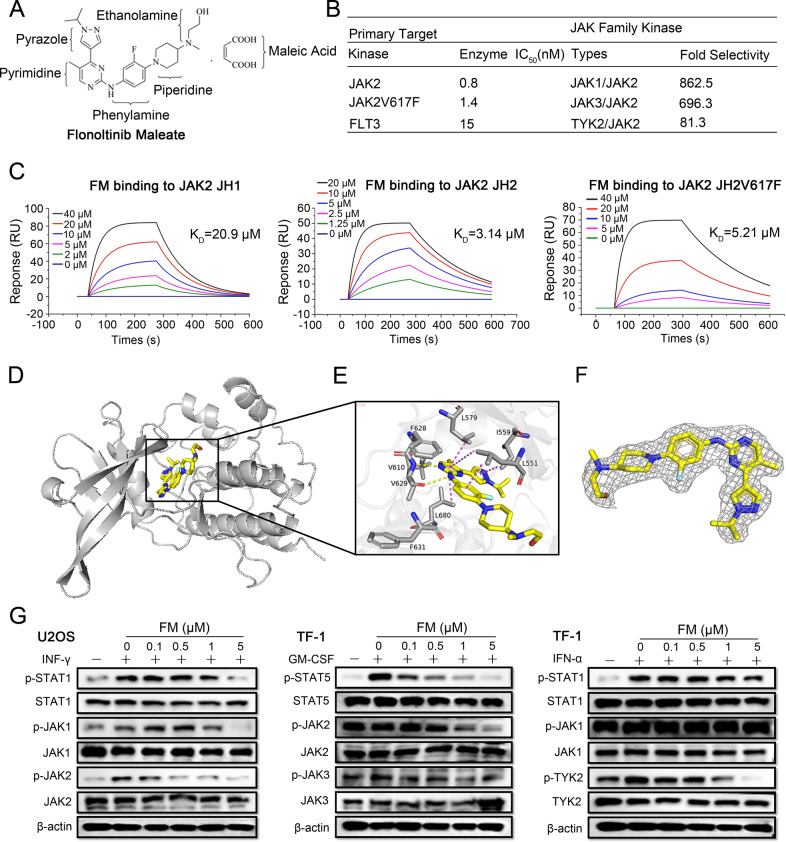
Chemical structure of FM and highly selective inhibition of JAK2 in JAK family. **A** Chemical structure of FM. **B** Selectivity profile of FM against JAK family. **C** Surface plasmon resonance assay for monitoring the affinity of JAK2 JH1, JAK2 JH2, and JAK2 JH2V617F with FM. **D** Cocrystal structure of JAK2-JH2 domain (cartoon) in complex with FM (sticks). **E** Magnified view between JAK2-JH2 and FM. Hydrogen bonds are represented by yellow dashed lines. **F** Electron densities of FM (2Fo-Fc, gray mesh, contoured at 3σ). **G** Identification of FM as a highly selective JAK2 inhibitor. Cells were preliminarily treated with FM for 1 h, prior to treatment with IFN-γ, GM-CSF, and IFN-α for an additional 20–60 min in U2OS and TF-1 cell lines, and analyzed via western blotting.

FM, a potent and highly selective JAK2 and FLT3 inhibitor, was designed and synthesized in our laboratory [35] (Fig. 1A). In in vitro kinase assays, FM showed strong kinase inhibition for JAK2, *JAK2*^V617F^, and FLT3 with the IC_50_ values of 0.8, 1.4, and 15 nM, respectively. FM showed 650–900 folds more selectivity to JAK2 than JAK1 and JAK3, and ~80 folds greater selectivity for JAK2 over TYK2 (Fig. 1B). Thus, FM exhibited the best selectivity for JAK2 over the JAK family among reported JAK2 inhibitors [36–39] (Table S2).

Next, we calculated the root-mean-square deviation (RMSD) values for the JAK2-JH1/JAK2-JH2 backbone atoms and non-hydrogen atoms of FM according to the structures obtained by molecular docking, and we also used MM/GBSA to calculate the absolute binding free energies for FM with JAK2 JH1 and FM with JAK2 JH2 systems. A detailed description of the results was provided in the SI (Fig. S1A–F and Tables S3–5). The $\Delta {{{\mathrm{G}}}}_{{{{\mathrm{bind}}}}}^{{{{\mathrm{cal}}}}}$ of FM/JAK2 is around −16.06 and −20.26 kcal/mol for JH1 and JH2, respectively. We could speculate that FM could simultaneously bind with JH1 and JH2 of JAK2, but the binding ability of FM on JAK2 JH2 is significantly stronger than JAK2 JH1. Then a surface plasmon resonance assay was used to compare the affinity of FM binding to JH1, JH2, and JH2V617F of JAK2. The results showed that FM could bind JAK2 JH2 and JAK2 JH2V617F protein with the KD values of 3.14 and 5.21 µM, respectively, indicating that FM had a better affinity for JAK2 JH2 compared to JAK2 JH1 and JAK2 JH2V617F (Fig. 1C). Considering these experimental results, we then focused on the interaction between JAK2 JH2 and FM. We obtained the crystal structure of the complex of JAK2 JH2 and FM (Fig. 1D–F and Table S6). Two nitrogen atoms of pyrimidine-2-amine in FM were found to form two hydrogen bonds with the carbonyl and NH backbone of V629 in the hinge region of JAK2. The hydrophobic interaction between FM and the surrounding amino acid residues of JAK2 could also be found in the complex of JAK2 JH2 and FM. The L551, I559, L579, and L680 of JAK2 could further enhance the binding stability between FM and JAK2 JH2. The benzene of FM could form a hydrophobic interaction with the side chain of L551. The I559 of JAK2 also interacted with the pyrazole ring of FM with hydrophobic interaction. In addition, the other hydrophobic residues near the active binding site also contributed to the binding affinity, such as the V610, F628, and F631 for JAK2 (Fig. 1D–F). The V617F mutation site is located above the N-terminus of the JAK2 JH2 domain; consistently, FM presented high inhibitory activity and selectivity for JAK2 JH2 protein.

To further determine the selectivity of FM to inhibit different JAK isotypes in cell lines, we performed a series of cytokine-stimulated cell-based assays [40, 41] and assessed the signal transduction of JAK subtypes. FM inhibited GM-CSF-induced p-STAT5, which involve JAK2/JAK2 signaling with an IC_50_ value of 0.12 µM. However, in JAK1/TYK2 pathway with IFN-α-induced p-STAT1, FM showed weak inhibition with an IC_50_ > 5 µM. In addition, in IFN-γ-induced p-STAT1 in U2OS cells and G-CSF-induced p-STAT3 in HEL cells which required the activity of JAK1/JAK2, the IC_50_ values for FM were 0.39 µM and 0.46 µM, respectively (Figs. 1G and S2A). These data indicated that the inhibitory activity of FM to JAK2 was greater than those of JAK1, JAK3, and TYK2, which was consistent with the high selectivity of FM in vitro kinase assays.

### In vitro cellular activity of FM

The activity of FM was evaluated on a panel of *JAK2*-dependent and *FLT3* mutated cell lines. As shown in Table S7, the anti-proliferative IC_50_ values of FM were <0.5 μM on *JAK2*^V617F^ mutant cell lines, and FM showed stronger anti-proliferative activity in mutant (Ba/F3-*JAK2*^V617F^) cell lines with an IC_50_ value of 0.20 ± 0.01 µM than that of wild-type cells (Ba/F3-*JAK2*^WT^) with an IC_50_ value of 0.39 ± 0.20 µM, which showed 1.95-fold better selectivity than *JAK2*^WT^ (V617F/WT ratio). The inhibitory effect of FM on *FLT3* mutant tumor cell lines was also obvious with an IC_50_ < 0.1 μM. Overall, FM showed significant anti-proliferative effects on multiple *JAK2* or *FLT3* mutant cell lines in vitro, and its effect was superior or comparable to those of ruxolitinib and fedratinib.

**Fig. 2:**
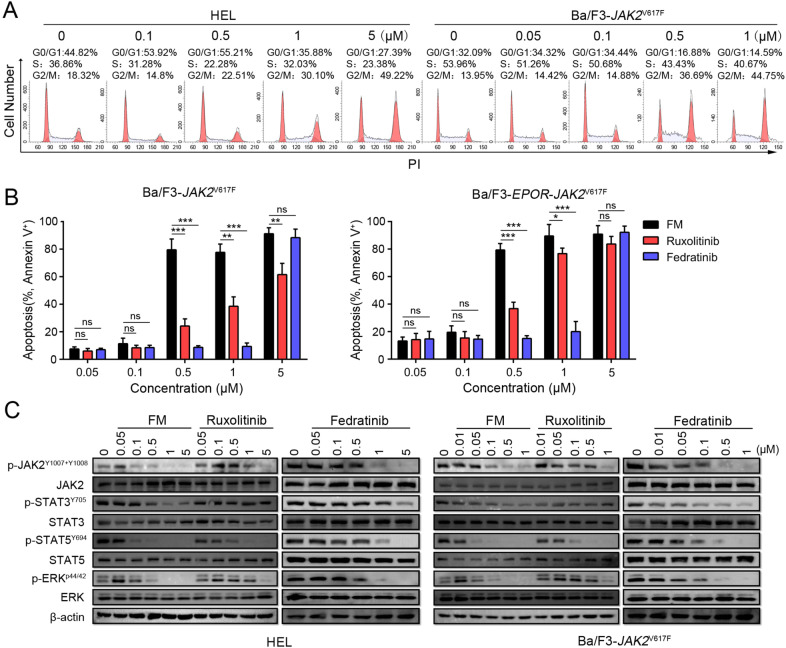
Inhibition of *JAK2*-dependent cell lines via FM in vitro. **A** HEL and Ba/F3-*JAK2*^V617F^ cells were supplemented with different concentrations of FM for 24 h, and the cell cycle was analyzed via propidium iodide (PI) staining. **B** Ba/F3-*JAK2*^V617F^ and Ba/F3-*EPOR*-*JAK2*^V617F^ cells were treated for 48 h with FM and analyzed by Annexin V and PI co-staining. Data are represented as mean ± SD, *p < 0.05, **p < 0.01, ***p < 0.001 vs. FM, *t*-test. **C** Effect of 3 h incubation with FM, ruxolitinib (Ruxo), and fedratinib (Fedra) on phosphorylation of JAK2, STAT3, STAT5, and ERK in HEL and Ba/F3-*JAK2*^V617F^ cell lines. β-actin served as the loading control.

To further evaluate the effects of *JAK2*^V617F^ or *FLT3* mutated cell lines, we treated Ba/F3-*JAK2*^V617F^, Ba/F3-*EPOR*-*JAK2*^V617F^, HEL, and MV-4-11 cells with serial levels of FM. The results showed that FM blocked the cell cycle at the G2-M phase (Figs. 2A and S2B–C) and induced tumor cell apoptosis in a concentration-dependent manner (Figs. 2B and S2D–E). In Ba/F3-*JAK2*^V617F^ and Ba/F3-*EROR*-*JAK2*^V617F^ cell lines, the activity of FM to induce apoptosis was significantly better than those of ruxolitinib and fedratinib in the low concentration range (Fig. 2B).

Aiming at the exploration of whether JAK2 inhibition by FM could affect the JAK/STAT pathway, phosphorylation of JAKs and downstream proteins were assessed in HEL and Ba/F3-*JAK2*^V617F^ cell lines. FM dose-dependently suppressed the phosphorylation of JAK2, STAT3, STAT5, and ERK1/2, and ruxolitinib and fedratinib served as positive controls (Fig. 2C). As FLT3 is another FM target, we also examined the phosphorylation of FLT3, as well as its downstream signaling effectors in the Molm-13 cell line. As expected, the levels of p-FLT3, p-STAT5, and p-ERK1/2 decreased significantly at low concentrations of FM (Fig. S2F). These results indicated that FM inhibited the phosphorylation of JAK2-directed signaling and triggered apoptosis in cell lines with JAK2 dependence. Thus, FM could possibly treat *JAK2*^V617F^ mutation-dependent diseases.

### FM exerting robust antitumor activity in Ba/F3-*JAK2*^V617F^ disease model

**Fig. 3:**
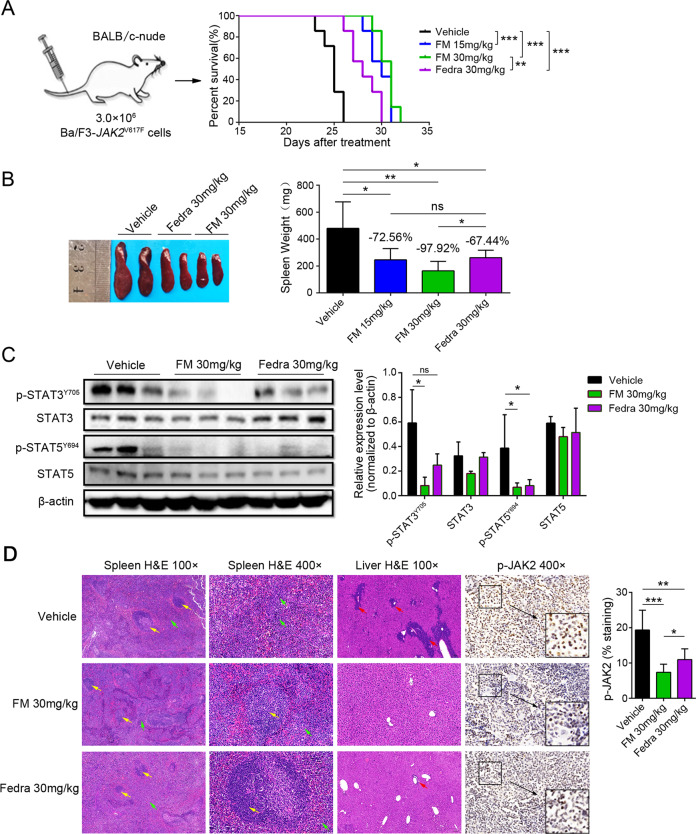
Effective inhibition of *JAK2*^V617F^-induced MPN mouse model in vivo by FM. BALB/c-nude mice received intravenous inoculation of 3.0 × 10^6^ Ba/F3-*JAK2*^V617F^-GFP cells and were administered with the vehicle, FM 15 and 30 mg/kg, and fedratinib 30 mg/kg bid. p.o. after 3 days, and the mice were executed after 22 days of treatment. **A** Kaplan–Meier analysis of survival between the vehicle-, FM- and fedratinib-treated groups was performed using the log-rank test (*n* = 7). **B** Spleens were acquired and analyzed (*n* = 6). **C** Western blot analysis of phosphorylated STAT3/5 and total STAT3/5 levels in spleens (*n* = 3). **D** Representative histological sections of the spleen and liver sections and the extent of myelo-erythroid infiltration were stained with H&E, and the expression of p-*JAK2* in the spleen was assayed via IHC staining. White pulp (yellow arrow), red pulp (green arrow), and tumor cell infiltration (red arrow) were marked. Images were obtained at ×100 and ×400 magnification. The histogram on the right panel is the quantitative statistics of IHC staining results performed by Image-Pro Plus. Data are represented as mean ± SD, *p < 0.05, **p < 0.01, ***p < 0.001 vs. vehicle, *t*-test.

To further investigate the effect of FM, we evaluated the in vivo efficacy in an MPN-like Ba/F3-*JAK2*^V617F^ mouse model, which was characterized by severe splenomegaly, extramedullary hematopoiesis, and reduced food intake. As shown in Fig. 3A, both FM and fedratinib significantly extended the survival, and the median survivals were respective 30 and 31 days in FM-treated (15 and 30 mg/kg) groups, and 28 days in fedratinib-treated (30 mg/kg) groups compared to 25 days in the vehicle group. In addition, FM markedly reduced the spleen weight, and the inhibition of the spleen growth was 72.56% and 97.92% in FM-treated (15 and 30 mg/kg) groups, and 67.44% in the fedratinib-treated group (Fig. 3B). There was no obvious body weight decrease at any dose of FM and fedratinib, and the animals were in good condition during treatment (Fig. S3A).

We then isolated spleen cells from mice with disease progression to assess the levels of crucial proteins. Similar to in vitro findings, FM decreased the p-STAT3 and p-STAT5 levels after 22 days of treatment (Fig. 3C). Immunohistochemistry (IHC) analysis indicated that both FM and fedratinib significantly inhibited the protein level of p-JAK2 in the spleen, whereas FM exhibited a stronger inhibitory effect than fedratinib (FM vs. fedratinib at 30 mg/kg, *p* = 0.0441) (Fig. 3D). Furthermore, the histopathological analysis revealed that the tumor infiltration was significantly reduced, and extramedullary hematopoiesis was improved in the FM therapy group. Unlike the myelo-erythroid infiltration observed in spleens of vehicle-treated mice, FM therapy restored normal splenic architecture (Fig. 3D). Overall, the expansion of the erythroid progenitor characteristic of the expression of *JAK2*^V617F^ was significantly reduced with FM therapy, which indicated that FM has the potential to treat the Ba/F3-*JAK2*^V617F^ disease model.

### Effects of FM in an *EPOR*-*JAK2*^V617F^-driven murine malignancy model

**Fig. 4:**
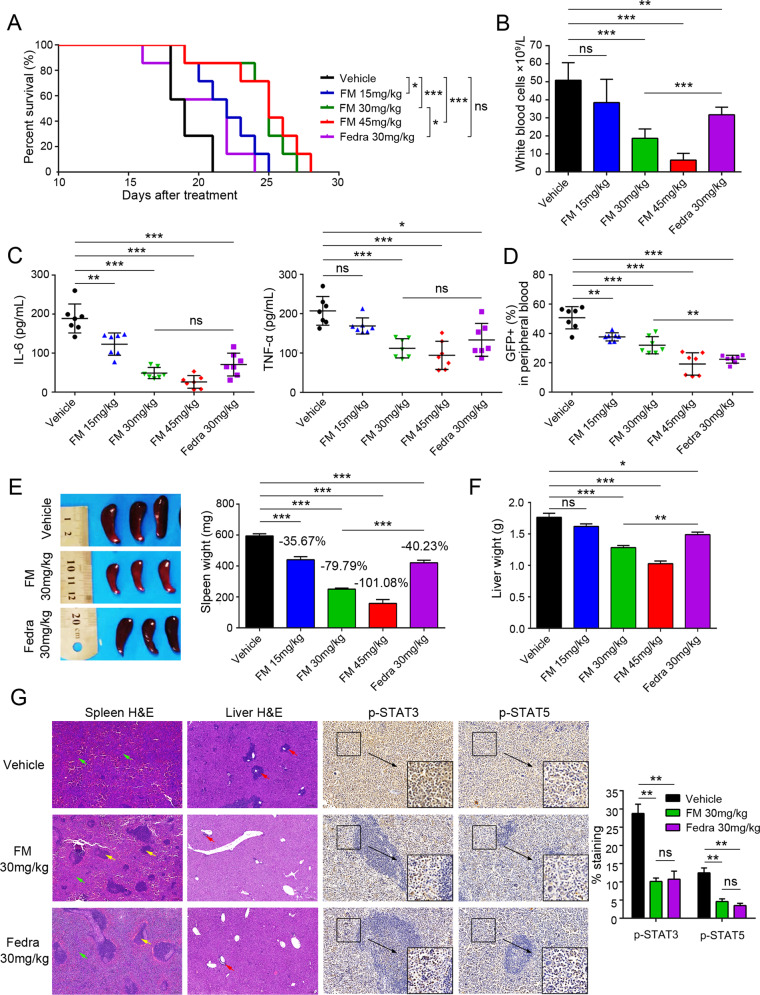
Efficacy of FM against Ba/F3-*EPOR*-*JAK2*^V617F^ malignancy mouse model. BALB/c-nude mice were intravenously injected with 1.0 × 10^6^ Ba/F3-*EPOR*-*JAK2*^V617F^–GFP cells and treated with vehicle, FM 15, 30, and 45 mg/kg and fedratinib 30 mg/kg bid. p.o. after 24 h, and the mice were sacrificed after 16 days of treatment. **A** Kaplan–Meier analysis of survival in Ba/F3-*EPOR*-*JAK2*^V617F^ mice in vehicle and FM or fedratinib treatment groups (*n* = 7). **B** White blood cell counts in PB were analyzed (*n* = 5). **C** Circulating IL-6 and TNF-α levels were analyzed in blood serum by ELISA (*n* = 7). **D** Fluorescence-activated cell sorting (FACS) analysis of the percentage of GFP^+^ cells in the PB at the end of the treatment (*n* = 7). **E** The size and weight of the spleen were acquired, and the spleen suppression rate was evaluated (*n* = 7). **F** Liver weights were analyzed (*n* = 7). **G** Splenic architecture and the extent of myelo-erythroid infiltration of the spleen and liver were observed in vehicle-treated animals compared to FM-treated animals. P-STAT3 and p-STAT5 levels were analyzed by IHC in the spleen. White pulp (yellow arrow), red pulp (green arrow), and tumor cell infiltration (red arrow) were marked. Images were obtained at ×200 magnification. The histogram on the right panel is the quantitative statistics of IHC staining results performed by Image-Pro Plus. Data are represented as mean ± SD, *p < 0.05, **p < 0.01, ***p < 0.001 vs. vehicle, *t*-test.

Next, we investigated the in vivo activity of FM using an *EPOR*-*JAK2*^V617F^-driven MPN mouse model, as the *EPOR* (erythropoietin receptor) gene activates JAK2 tyrosine kinase [42, 43]. Compared to the Ba/F3-*JAK2*^V617F^ disease model, this model displayed a greater tumor burden, faster onset, and more severe hepatosplenomegaly. Consistent with the results of the previous model, although the *EPOR*-*JAK2*^V617F^-driven malignancy mouse model progressed rapidly, FM still showed a good therapeutic effect. Both FM and fedratinib significantly prolonged the survival of tumor-bearing mice compared to the vehicle. The median survival times were 22, 25, and 25 days in the FM-treated (15, 30, and 45 mg/kg) groups and 22 days in the fedratinib-treated (30 mg/kg) group versus 19 days in the vehicle group, respectively (Fig. 4A). Complete blood count analysis showed that FM and fedratinib reduced the white blood cells at the same dose of 30 mg/kg (Fig. 4B). We also observed that the levels of inflammatory cytokines, IL-6 and TNF-α, decreased markedly after FM treatment in a dose-dependent manner (Fig. 4C). Evaluation of the tumor burden in PB showed that FM effectively inhibited Ba/F3-*EPOR*-*JAK2*^V617F^ cell proliferation (Fig. 4D).

In addition, vehicle-treated mice showed enlargement of the spleen and liver with average weights of 594.67 ± 36.58 mg and 1.76 ± 0.17 g, respectively. FM- and fedratinib-treated mice were remarkably inhibited in terms of spleen and liver, and mice almost recovered to normal weights at FM of 45 mg/kg (Fig. 4E–F). As shown in Fig. 4G, the histology of the spleens from mice in the vehicle group showed extensive infiltration with extramedullary hematopoiesis and complete effacement of splenic architecture, whereas there was a partial restoration of the splenic architecture in the high-dose group with intact follicles covering 30%–50% of the area. In addition, the liver histology revealed mild infiltration with extramedullary hematopoiesis, ranging from small periportal aggregates and scattered megakaryocytes to large perivascular and random aggregates of maturing granulocytes in the vehicle and low-dose groups, but not in the high-dose group (Fig. 4G). The tissues of FM treatment mice did not show serious damage (data not shown), which indicated that FM did not cause obvious visceral toxicity. Moreover, we analyzed the levels of p-JAK2, p-STAT3, and p-STAT5 in the spleen via IHC and showed that the phosphorylation levels of downstream proteins were inhibited by FM treatment (Figs. 4G and S3B). Overall, these results indicated that FM exerted robust activity in the Ba/F3-*EPOR*-*JAK2*^V617F^ disease model in vivo.

### PK/PD studies of FM in Ba/F3-*EPOR*-*JAK2*^V617F^-driven mouse model

**Fig. 5:**
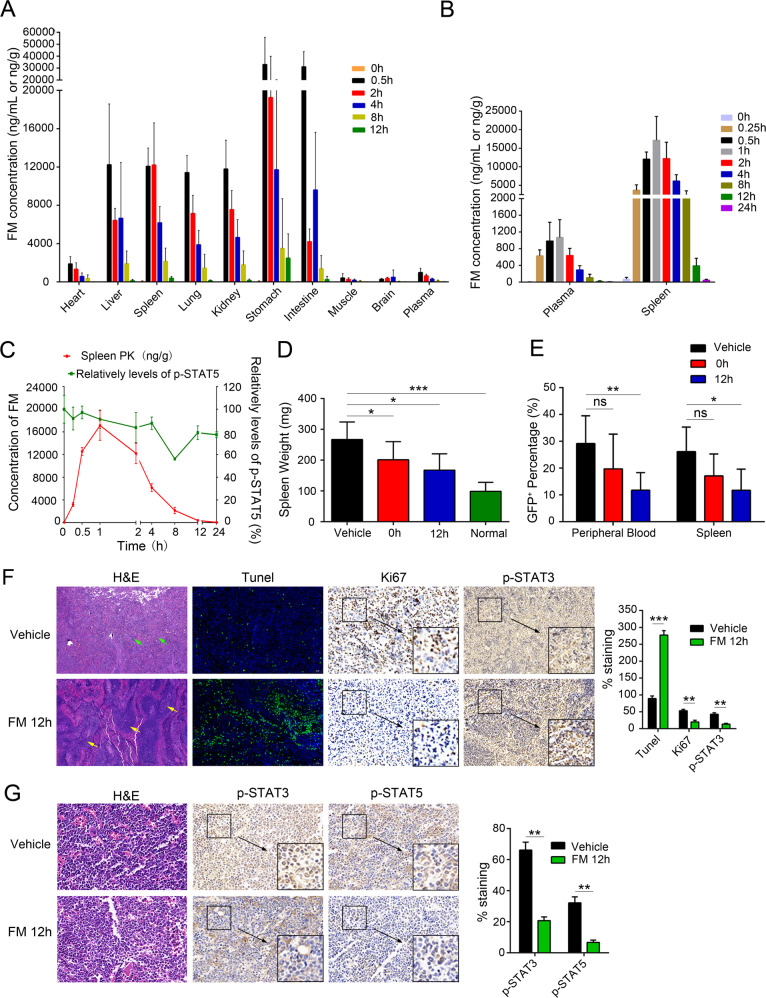
Pharmacokinetics and pharmacodynamics of FM in the Ba/F3-*EPOR*-*JAK2*^V617F^ disease model. All mice were given FM 30 mg/kg, and 10 days after inoculation, mice were randomized into 8 groups (*n* = 6) and sacrificed at the indicated time points (0–24 h) after the last FM 30 mg/kg by oral gavage. **A**–**B** FM concentrations in different organizations in Ba/F3-*EPOR*-*JAK2*^V617F^ bearing mice were determined using LC-MS/MS (*n* = 6). **C** Concentration-time curve and pharmacodynamic time curve of FM in the spleen (*n* = 3). **D** FACS analysis of the tumor burden in PB and spleen at 0 and 12 h after the last FM 30 mg/kg oral administration of FM (*n* = 3). **E** Mice were sacrificed, and the spleen was weighed at 0 and 12 h (*n* = 6). **F** Spleens were obtained for H&E, TUNEL, Ki67, and IHC of p-STAT3 and p-STAT5 staining after 12 h of FM treatment compared to vehicle treatment. White pulp (yellow arrow) and red pulp (green arrow) were marked. Images were obtained at ×200 magnification. **G** BM was analyzed for H&E and IHC of p-STAT3 and p-STAT5 staining after 12 h of FM treatment compared to vehicle treatment. The histogram on the right panel is the quantitative statistics of TUNEL, Ki67, and IHC staining results performed by Image-Pro Plus. Data are represented as mean ± SD, *p < 0.05, **p < 0.01, ***p < 0.001 vs. vehicle, *t*-test.

The PK/PD studies of single and repeated administration of FM were examined in a Ba/F3-*EPOR*-*JAK2*^V617F^ malignancy mouse model, and the findings were displayed in SI (Figs. 5A–G, Fig. S4A–F, Table 1, and Tables S8–9). FM significantly inhibited tumor progression in Ba/F3-*EPOR*-*JAK2*^V617F^ tumor-bearing mice with high drug exposure in tumor-bearing tissues (Fig. 5A–B). At 8 and 12 h after the last administration, the FM concentration in the spleen was 2151.00 ± 1385.33 ng/g and 390.80 ± 181.44 ng/g, respectively, and the exposure AUC_(0–24h)_ and C_max_ of the spleen were 17.95 and 14.14 folds higher than that of plasma, respectively, which indicated that FM was rapidly eliminated in the plasma and accumulated in the spleen (Table 1). Furthermore, as the spleen and BM are both target tissues of MPNs, we analyzed the histology and the key proteins of BM. As shown in Fig. 5G, FM therapy reduced myelo-erythroid infiltration and caused p-STAT3 and p-STAT5 downregulation at 12 h from the last administration. These results further illustrated the potential of FM to suppress disease progression with decreased systemic toxicity.

**Table 1:** Pharmacokinetic parameters of plasma and spleen in Ba/F3-*EPOR*-*JAK2*^V617F^ tumor-bearing mice after single or repeated oral administration of FM.

Pharmacokinetics	Single administration		Repeated administration	
	Plasma	Spleen	Plasma	Spleen
C_max_ (ng/mL)	1028.18 ± 173.54	12009.71 ± 3473.35	1283.41 ± 454.89	18145.99 ± 5679.92
AUC_0–24 h_ (ng^.^h/mL)	2990.15 ± 557.79	45706.7 1 ± 9606.67	3742.84 ± 703.74	67200.44 ± 11858.88
T_1/2_ (h)	2.31 ± 0.79	2.71 ± 0.51	2.85 ± 1.14	2.37 ± 0.58
Vdss (L/kg)	32.84 ± 10.12	2.67 ± 0.90	32.81 ± 11.21	1.52 ± 0.23
Cl (L/h/kg)	10.12 ± 2.17	0.68 ± 0.15	8.23 ± 1.91	0.46 ± 0.09

At 12 h following administration, the tumor burden in the plasma and spleen was the lowest (Fig. 5D), whereas the FM concentration was the highest in the spleen (Fig. 5B). There was no significant difference in FM exposure AUC_(0–24 h)_ in the plasma and tissues between single and repeated administration of FM, implying that the repeated administrations of FM did not cause accumulation in the body and toxicity (Table 1). Moreover, the bioavailability of FM in rats and dogs was nearly 60%, whereas it was found to be 19% in rats and 37% in dogs for fedratinib (data not shown). These results illustrated the rationality and feasibility of twice-daily administration of FM and showed good PK/PD characteristics and low toxicity.

### Efficacy of FM in *JAK2*^V617F^-induced BMT myelofibrosis model

**Fig. 6:**
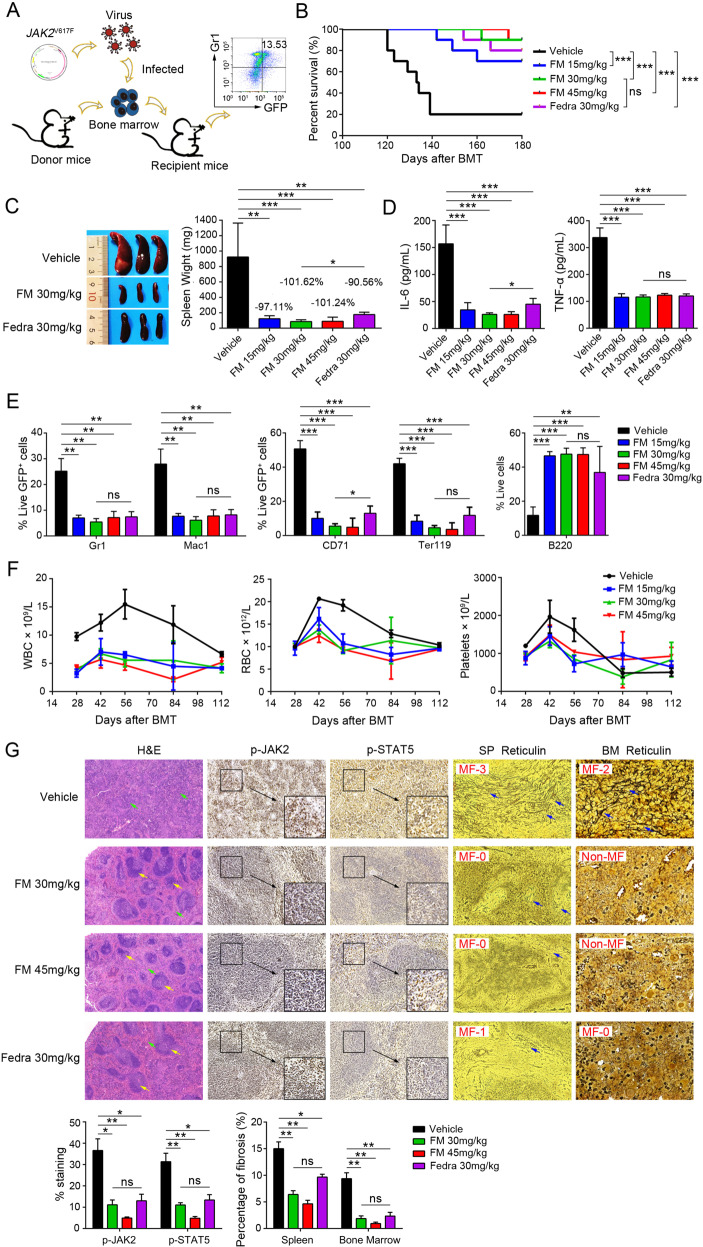
FM showing efficacy against *JAK2*^V617F^ bone marrow transplantation (BMT) mouse model of myelofibrosis in vivo. BMT mice were treated with vehicle, FM 15, 30, and 45 mg/kg and fedratinib 30 mg/kg bid. p.o. after 14 days. **A** Schematics of the method for engraftment of BM cells infected with *JAK2*^V617F^ mutation virus into irradiated BALB/c mice and FACS monitoring of disease progression. Each donor mouse sample was injected into three recipient mice. **B** Kaplan–Meier survival curve in the *JAK2*^V617F^ model of myelofibrosis after long-term treatment with FM. Statistical significance in survival between the vehicle and FM groups was evaluated using the log-rank test (*n* = 10). **C** The spleen weight was examined (*n* = 6). **D** Effects of FM and fedratinib on IL-6 and TNF-α concentrations in serum of tumor-bearing mice were measured via ELISA (*n* = 4). **E** Mice were executed and splenocytes were stained using fluorescently conjugated antibodies specific for Gr1 (granulocytes), Mac1 (macrophages), CD71 (early erythroid progenitors), Ter119 (late erythroid progenitors), and B220 (B cells) (*n* = 3). **F** White blood cell count, red blood cell count, and platelet count of recipient mice were monitored (*n* = 6). **G** Representative images of histological tumor sections are shown. The spleen was stained with H&E and IHC of p-JAK2 and p-STAT5. BM was stained with Gomori ammonia silver. White pulp (yellow arrow) and red pulp (green arrow) were marked. Images were obtained at ×100 or ×400 magnification. The histogram on the lower panel is the quantitative statistics of IHC and fibrosis staining results performed by Image-Pro Plus. Data are represented as mean ± SD, *p < 0.05, **p < 0.01, ***p < 0.001 vs. vehicle, *t*-test.

To further assess the therapeutic efficacy of FM in primary MF, we employed a *JAK2*^V617F^-induced primary MF mouse model (Fig. 6A), which could imitate the pathogenesis of patients with clinical MF. On day 14 post-BMT, 13.53 ± 2.64% of GFP^+^Gr1^+^ cells were examined in PB (Fig. S5A). Subsequently, the mice were randomized into five groups and received different doses of FM or fedratinib treatment. As the treatment progressed, GFP^+^ Gr1^+^ cells were significantly reduced (Fig. S5C). The median survival time of mice was >180 days in FM-treated (15, 30, and 45 mg/kg) groups and fedratinib-treated (30 mg/kg) group *vs*. 133.5 days in the vehicle group (Fig. 6B). Although there was no significant difference between FM and fedratinib in the median survival time of mice in the 30 mg/kg groups, FM exhibited a stronger therapeutic effect on splenomegaly than fedratinib at the same dose (*p* = 0.0158) (Fig. 6C). The splenomegaly was dose-dependently inhibited, and the inhibition of splenomegaly was 97.11%, 101.62%, and 101.24% at FM doses of 15, 30, and 45 mg/kg, respectively, whereas it was 90.56% in the fedratinib 30 mg/kg group (Fig. 6C). The spleen of mice treated with FM 30 mg/kg recovered to the normal level. In addition, compared with the vehicle group, the levels of IL-6 and TNF-α in PB of mice were significantly and dose-dependently suppressed by FM treatment (Fig. 6D).

To determine the effects of FM on various hematopoietic cell compartments and differentiation stages, we then analyzed the tumor burden in the spleen (Fig. 6E) and BM (Fig. S5D). FM exhibited a noticeable reduction in the proportion of granulocytes (Gr1^+^), macrophages (Mac1^+^), GFP^+^CD71^+^ cells (early erythroid progenitors), and GFP^+^Ter119^+^ cells (late erythroid progenitors) compared to the vehicle mice, while the proportion of B220^+^ cells (B cells) increased, reaching the level of normal mice (Figs. 6E and S5D). *JAK2*^V617F^ BMT mice induced an increase in WBCs and HCT, but after FM treatment, the number of WBCs and HCT decreased (Figs. 6F and S5B). These data revealed that FM was effective in various hematopoietic cell compartments.

Further analysis of the spleen of the vehicle mice showed that a large number of tumor cells infiltrated into the red pulp, and the dense tissue structure was destroyed in the vehicle group (Fig. 6G). However, after FM treatment, tumor cell infiltration in the spleen was repressed in a dose-dependent manner, and the spleen structure gradually returned to the normal tissue structure. The IHC results showed that p-JAK2 and p-STAT5 were significantly inhibited by FM. Importantly, we observed severe reticular fibrosis (black filamentous substance) in the spleen and BM of mice in the vehicle group. As shown in Fig. 6G, the vehicle-treated group were myelofibrosis-3 (MF-3) in the spleen and MF-2 in BM, respectively, based on BM fibrosis grading criteria of the World Health Organization (WHO) [44]. FM treatment effectively inhibited the progression of reticular fibrosis and almost completely inhibited the MF progress at FM 30 and 45 mg/kg with non-MF in BM. Both FM and fedratinib could effectively inhibit reticular fibrosis in the spleen and BM, and there was no significant difference between FM and fedratinib at the same dose (Fig. 6G). To further understand the effect of FM on normal mice and normal human CD34^+^ cells, we conducted a further analysis on CD34^+^ cells and the results indicated that FM may have potential myelosuppressive effects (Fig. S6A–B). Overall, these findings indicated that FM was highly effective in the *JAK2*^V617F^ BMT myelofibrosis model and had no significant toxicity during long-term treatment.

### Effects of FM on erythroid progenitor cell growth and JAK2 signaling of primary *JAK2*^V617F^ cells from patients with MPNs

**Fig. 7:**
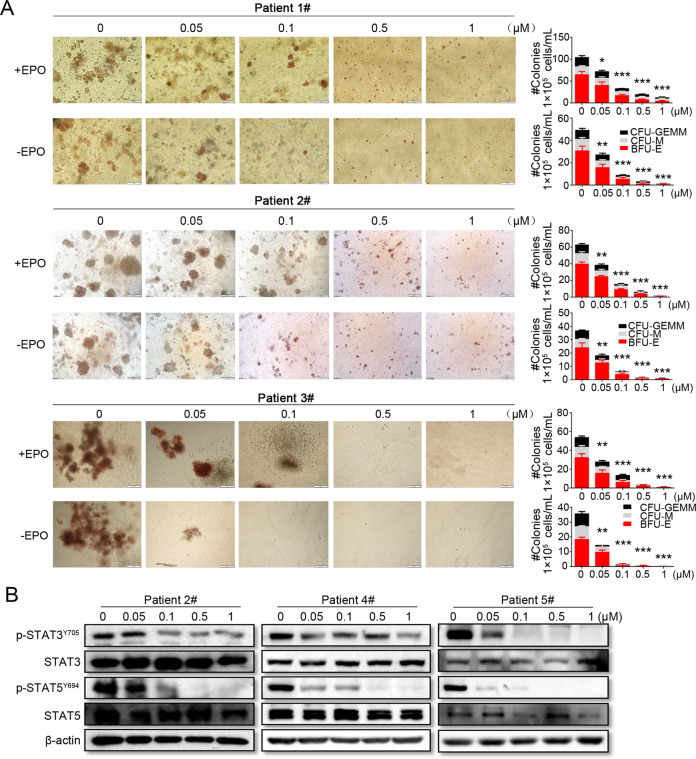
Effects of FM on erythroid colony formation and JAK2/STAT signaling in MPN patient samples with activating *JAK2*^V617F^ mutation. **A** Mononuclear cells were isolated from the PB or BM of patients with MPNs (Patient Number: 1#, 2#, and 3#) and incubated with FM in methylcellulose-based media. Hematopoietic colony-forming capacity was calculated by the total number of BFU-E, CFU-M, and CFU-GEMM on day 14. **B** MPN patient cells (Patient Number: 2#, 4#, and 5#) were incubated with various concentrations of FM for 4 h, and the phosphorylation of STAT3 and STAT5 were analyzed via western blot analysis. Data are represented as mean ± SD, *p < 0.05, **p < 0.01, ***p < 0.001 vs. vehicle, *t*-test.

To investigate the efficacy of FM on primary cells acquired from the patients with MPNs carrying *JAK2*^V617F^ mutation, colony formation assays were conducted using mononuclear cells acquired from the PB or BM of patients with MPNs. The features of patients with MPNs are described in Table S1. FM showed a stronger inhibitory effect on burst-forming unit-erythroid (BFU-E), and it also inhibited colony-forming unit-megakaryocytes (CFU-M) and colony-forming unit-granulocytes, erythrocytes, monocytes, and megakaryocytes (CFU-GEMM) compared with the non-treatment group (Fig. 7A). To explore the impact of erythropoietin (EPO) on colony formation, colony formation repression via FM was examined in an EPO-independent medium. In the EPO-free medium, the inhibitory effect of FM on colonies was more obvious than that with EPO medium in patients with MPNs and *JAK2*^V617F^ mutation (Fig. 7A).

We next assessed the expression of p-STAT3/5 in patients 2#, 4#, and 5#, and FM showed robust inhibition in JAK-STAT signaling (Fig. 7B). These results indicated that FM had a potent therapeutic effect on primary *JAK2*^V617F^ cells from patients with MPNs.

## Discussion

The JAK/STAT signaling pathway participates in cell proliferation, differentiation, apoptosis, and immune regulation [45, 46], and STATs are direct substrates of JAKs, which can transmit signal agents to the nucleus and regulate the expression of specific genes [47, 48]. Hyperactivation of JAK2 triggered by the *JAK2*^V617F^ mutation leads to MPNs [49, 50]. As marketed drugs for the treatment of MPNs, ruxolitinib is a JAK1/JAK2 inhibitor, and fedratinib is a JAK2/FLT3 inhibitor. Both of them bind to the JAK2 JH1 domain, which may be the reason why their selectivity is not sufficiently high, and they cause significant side effects [32, 33]. *JAK2*^V617F^ mutation is located in the JAK2 JH2 domain. Thus, targeting JAK2 JH2 is more conducive to the treatment of MPNs [27, 51].

Through extensive studies on the binding mode of the dual JAK2/FLT3 inhibitor pacritinib, it was found that the 4-substituent pyrimidine-2-amine pharmacophore of pacritinib was crucial to maintain the ATP domain binding group for JAK2. According to the computer-aided drug design instruction, we designed pyrazole instead of phenyl to increase the enzymatic activity for JAK2/FLT3 and also introduced a series of hydrophile groups in the benzene ring to improve the oral bioavailability. After structure-activity relationship discussion, metabolic stability, in vitro and in vivo activity screening, and toxicity evaluation, we finally selected the compound FM as a clinical candidate compound.

Our study has demonstrated the high selectivity of FM for JAK2 over other JAK isotypes. It has been known that the marketed JAK2 inhibitors such as ruxolitinib and fedratinib bind to the ATP site of the catalytic domain JAK2 JH1 [52]. However, FM is different from previous JAK2 inhibitors because of its unique ability to selectively bind to the JH2 domain of JAK2, exerting its function through an allosteric mechanism. FM has a stronger affinity for JH2 than for JH1 of JAK2, and the crystal structure of the complex JAK2 JH2 and FM showed that FM could stably bind to the JAK2 JH2 protein. Therefore, FM may avoid the high effect inhibition of other JAK subtypes and improve the selectivity of JAK2.

The antitumor activity of FM on *JAK2* and *FLT3* mutant cell lines showed that the anti-proliferative IC_50_ of FM was better than that of ruxolitinib and fedratinib. The p-JAK2, p-STAT5, p-STAT3, and p-ERK protein levels were significantly downregulated by FM in both *JAK2* and *FLT3* mutated cell lines. Moreover, FM could significantly inhibit the growth of erythroid progenitor cells and JAK2 signaling in the primary cells of patients with *JAK2*^V617F^ mutations.

In *JAK2*^V617F^-driven MPN models, including Ba/F3-*JAK2*^V617F^ and Ba/F3-*EPOR*-*JAK2*^V617F^ disease models, oral FM at doses of 15, 30, or 45 mg/kg twice a day could dose-dependently inhibit splenomegaly and improve survival. FM suppressed spleen and BM fibrosis and significantly inhibited the development of tumor cells in the *JAK2*^V617F^ BMT mouse model. Compared with other JAK2 inhibitors, FM had a stronger affinity for the pseudokinase domain JH2 than JH1 of JAK2, and the bioavailability of FM in rats and dogs was nearly 60% as compared to 19% in rats and 37% in dogs of fedratinib. Therefore, the therapeutic effect of FM in these models was better than that of other JAK2 inhibitors. The PK/PD characteristics of FM indicate that AUC_(0–24h)_ and C_max_ exposed to the spleen are greater than that of plasma and are less distributed in the brain, suggesting that it has the potential to suppress disease progression and reduce systemic toxicity.

In summary, FM displays potent efficacy in vitro and in vivo on MPNs and possesses favorable PK/PD characteristics and low toxicity. Therefore, our preclinical study supports that FM could be a potent candidate drug for patients with JAK2-driven MPN with reduced adverse effects and better treatment outcomes than ruxolitinib and fedratinib.

### Supplementary information

Supplementary Infomation

Supplementary Table

Supplementary Figure 1

Supplementary Figure 2

Supplementary Figure 3

Supplementary Figure 4

Supplementary Figure 5

Supplementary Figure 6
