# Correction to: Preclinical studies of Flonoltinib Maleate, a novel JAK2/FLT3 inhibitor, in treatment of *JAK2*^V617F^-induced myeloproliferative neoplasms

**DOI:** 10.1038/s41408-024-01058-y

**Published:** 2024-06-26

**Authors:** Mengshi Hu, Tao Yang, Linyu Yang, Lu Niu, Jinbing Zhu, Ailin Zhao, Mingsong Shi, Xue Yuan, Minghai Tang, Jianhong Yang, Heying Pei, Zhuang Yang, Qiang Chen, Haoyu Ye, Ting Niu, Lijuan Chen

**Affiliations:** 1 State Key Laboratory of Biotherapy and Cancer Center, National Clinical Research Center for Geriatrics, West China Hospital of Sichuan University, Chengdu, China; 2 Department of Hematology and Research Laboratory of Hematology, West China Hospital of Sichuan University, Chengdu, China; 3 Chengdu Zenitar Biomedical Technology Co., Ltd, Chengdu, China

**Keywords:** Myeloproliferative disease, Targeted therapies

Correction to: *Blood Cancer Journal* (2022) 12:37 10.1038/s41408-022-00628-2, published online 07 March 2022

Following the publication of this article, errors were noted in Figures 4G and 7A. Upon reviewing the original data, it was realized that H&E staining for the Fedra 30mg/kg in the Ba/F3-*EPOR*-*JAK2*^V617F^ malignancy mouse model (Fig. 4G) was inaccurately analyzed as those from the *JAK2*^V617F^-induced MPN mouse model (Fig. 3D). This resulted in reuse of data from the Fedra 30mg/kg group. The corrected figure is presented below.

In Figure 7A, the image labeled 0.05 μM for Patient 2# is incorrect. Upon comparing with the original data, an error was determined due to the fact that 2 images were captured for each concentration. The data marked as 0 μM was mistakenly used in place of the 0.05 μM data during organization. The accurate data for Patient 2# is presented below.

The authors confirm these changes have no impact on the conclusions of the study and apologize for any inconvenience caused by these errors.
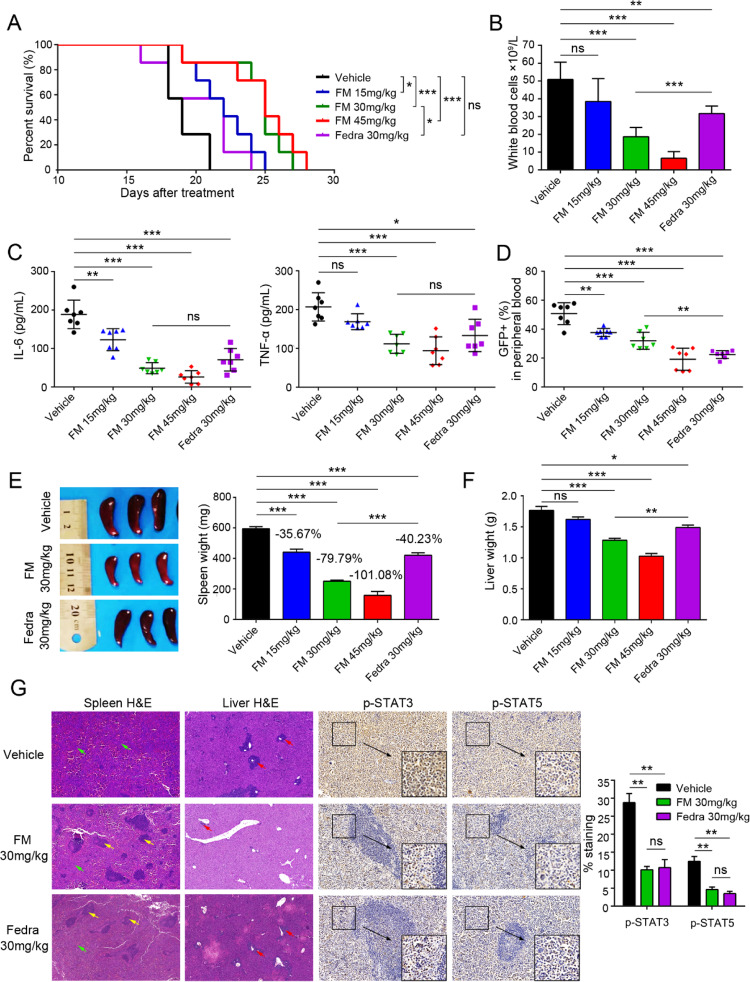


**Fig. 4** Efficacy of FM against Ba/F3-*EPOR*-*JAK2*^V617F^ malignancy mouse model. BALB/c-nude mice were intravenously injected with 1.0 × 10^6^ Ba/F3-*EPOR*-*JAK2*^V617F^ –GFP cells and treated with vehicle, FM 15, 30, and 45 mg/kg and fedratinib 30 mg/kg bid. p.o. after 24 h, and the mice were sacrificed after 16 days of treatment. **A** Kaplan-Meier analysis of survival in Ba/F3-*EPOR*-*JAK2*^V617F^ mice in vehicle and FM or fedratinib treatment groups (*n* = 7). **B** White blood cell counts in PB were analyzed (*n* = 5). **C** Circulating IL-6 and TNF-α levels were analyzed in blood serum by ELISA (*n* = 7). **D** Fluorescence-activated cell sorting (FACS) analysis of the percentage of GFP^+^ cells in the PB at the end of the treatment (*n* = 7). **E** The size and weight of the spleen were acquired, and the spleen suppression rate was evaluated (*n* = 7). **F** Liver weights were analyzed (*n* = 7). **G** Splenic architecture and the extent of myelo-erythroid infiltration of the spleen and liver were observed in vehicle-treated animals compared to FM-treated animals. P-STAT3 and p-STAT5 levels were analyzed by IHC in the spleen. White pulp (yellow arrow), red pulp (green arrow), and tumor cell infiltration (green arrow) were marked. Images were obtained at ×100 and ×200 magnification. The histogram on the right panel is the quantitative statistics of IHC staining results performed by Image-Pro Plus. Data are represented as mean ± SD, *p < 0.05, **p < 0.01, ***p < 0.001 vs. vehicle, *t*-test.
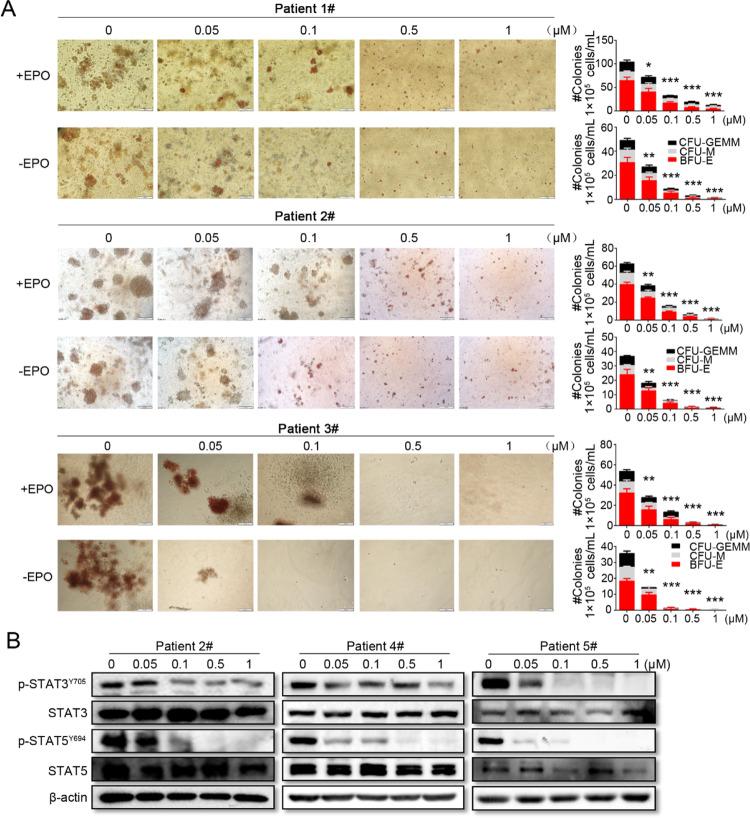


**Fig. 7** Effects of FM on erythroid colony formation and JAK2/STAT signaling in MPN patient samples with activating *JAK2*^V617F^ mutation. **A** Mononuclear cells were isolated from the PB or BM of patients with MPNs (Patient Number: 1#, 2#, and 3#), and incubated with FM in methylcellulose-based media. Hematopoietic colony-forming capacity was calculated by the total number of BFU-E, CFU-M, and CFU-GEMM on day 14. **B** MPN patient cells (Patient Number: 2#, 4#, and 5#) were incubated with various concentrations of FM for 4 h, and the phosphorylation of STAT3 and STAT5 were analyzed via western blot analysis. Data are represented as mean ± SD, *p < 0.05, **p < 0.01, ***p < 0.001 vs. vehicle, *t*-test.

