# Supplementary material for: Preclinical studies of Flonoltinib Maleate, a novel JAK2/FLT3 inhibitor, in treatment of JAK2V617F-induced myeloproliferative neoplasms
Source: Blood Cancer J. 2022 Mar 7;12(3):37. doi: 10.1038/s41408-022-00628-2 (PMC8901636; doi:10.1038/s41408-022-00628-2)
Supplement: Supplementary file 1 — Supplementary Infomation [file 41408_2022_628_MOESM1_ESM.docx]

**Supplementary Infomation**

# Supplementary materials and methods

## Compounds

For FM, Ruxilitinib and Fedratinib, in the *in vitro* test, stock solutions were prepared in DMSO, with final dimethyl sulfoxide concentration of 0.1% in cell-based assays. For *in vivo* studies, dosing solutions were prepared in 0.5% methylcellulose (w/v) and 0.1% Tween-80 in H_2_O (MC/Tween) for oral administration.

## Molecular Docking Study

The full JAK2 protein (UniProt ID: O60674) consisted of 1132 amino acids, and composed of four domains: a FERM domain near the N-terminal (FERM), a SH2 domain (SH2), a pseudokinase domain (JH2) and a kinase domain (JH1). The inhibitors can target the JH2 or/and JH1 domain from the protein database bank (PDB). Therefore, in this work, we will construct the complex systems for FM with JAK2-JH1 and JAK2-JH2. Therefore, the receptor protein JAK2-JH1 (PDB ID: 4IVA) and JAK2-JH2 (PDB ID: 6BSO) were selected as the docking model. The protein structure was processed via PyMOL[1] to remove the solvent molecules and the origin bound ligand. The protein structure was pretreated with the AutoDockTools[2], including adding hydrogen, equilibrium charge, adjustment of unreasonable atomic overlap, and so on. The molecular structure of FM was constructed using BIOVIA Draw 2021 software. The molecule structures were minimized by PM3 force field[3] by MOPAC 2016[4]. The partial charges for receptor and ligands were assigned using AutoDockTools with Gasteiger method[5]. A grid map of 40 ×40 ×40 point with 0.375 Å grid spacing was generated using AutoGrid module[6] based on the center of origin compound within protein active pocket. The 100 conformations of each system were finally generated for 150 population size with 2,500,000 maximum number of energy evaluation for each docking experiment using AutoDock 4.2.6 [6]. Subsequently, the best conformation of each docking models was remained. The “re-docking” strategy was used to assess the docking power via the used crystal structures. To this point, we then applied molecular docking method in the construction of FM/JAK2-JH1 and FM/JAK2-JH2 complex structures.

## Molecular Dynamics Simulation

The complex structures of FM/JAK2-JH1 and FM/JAK2-JH2 obtained from the docking study (Figure S1). The AMBER ff19SB force field [7] was used to create topology parameters of JAK2 protein. To correctly describe the FM force field, the general Amber force field (GAFF) [8] generation procedure was used. The geometry structure was optimized at B3LYP/6-31G level of theory using Gaussian 09[9]. To obtain the partial atomic charges, the restrained electrostatic potential (RESP) protocol[10] was employed at the HF/6-31G^*^ level of theory. The force field parameters were generated using the Antechamber module. Firstly, the complex systems were dissolved in a TIP3P water[11] with cuboid box and the systems were neutralized using the Cl^-^ or Na^+^ ions. To reduce the effect of unfavorable interactions produced by solvents and ion, we used two steps to minimize the complex systems for the two ligand/JAK2 complex systems. Following, the system was heated to 300 K and kept the pressure to 1 bar. Subsequently, the whole system underwent the 100 ns molecular dynamics for final data collection and analyses. The periodic boundary conditions were applied to avoid edge effects and a cut-off radius of 12 Å was employed for van der Waals interactions. The particle mesh Ewald (PME) algorithm[12] also was used in calculating the long-range electrostatic interactions. The SHAKE algorithm[13] was used to constrain the covalent bond involved with hydrogen atoms. The detail of the molecular dynamic simulation can be found in the other references [14-15]. The result was analyzed by the *CPPTRAJ* [16-18].

## Binding Free Energy

To quantitatively assess the binding affinity of FM with JAK2-JH1 and JAK2-JH2, it would be useful to calculate the corresponding binding free energies. In this work, molecular mechanics generalized Born surface area (MM/GBSA)[19-20] were employed to calculate the binding free energies. MM/GBSA is represented by the following equations:

$\Delta G_{binding}=\Delta G_{complex}-\Delta G_{protein}-\Delta G_{ligand}$ (1)

$G=E_{gas}+G_{sol}-TS$ (2)

$E_{gas}=E_{int}+E_{vdW}+E_{ele}$ (3)

$G_{sol}=G_{el}+G_{nonel}$ (4)

where $\Delta G_{complex}$, $\Delta G_{protein}$, and $\Delta G_{ligand}$ indicate the free energies for complex, protein, and ligand. MM/GBSA was used to calculate these values based on the statistical average obtained from one MD trajectory. The solvation free energy ($G_{sol}$) comprises electrostatic ($G_{el}$) and non-electrostatic ($G_{nonel}$) terms. $G_{nonel}$ represents the combined effects of the unfavorable cost of surface formation and favorable van der Waals (vdW) interactions between solute and solvent. This factor was evaluated by using the equation *γ = SA* + *b*, where *γ* = 0.0072 kcal/Å^2^ and *b* = 0.0 kcal/mol. The solvent-accessible surface area (*SA*) was estimated by using LCPO[21]. $G_{el}$ was calculated using the Generalized Born (GB) equation[22-23]. The interior of the system was assumed to be filled with dielectric constant (ε) of 1. The solvent water considered had a high dielectric constant (ε = 80). The gas-phase energy contributions ($E_{gas}$) were calculated using *mmpbsa_py*_*energy* according to the force field with mbondi2 for PBRadii[24]. For each complex system, the binding energies were averaged over 1,000 frames of the last 20 ns MD trajectory. Entropy contributions to the binding free energy were added as a further refinement and averaged for a 0.2 ns interval of the last 20 ns of MD. The entropy was estimated based on the changes in the degrees of freedom including translation, rotation, and vibration[25]. The free energy was calculated by using *MMPBA.py* [20].

## Western blot analysis

Cells were incubated with compounds for 3 hours, and performed according to standard methods. p-JAK2 (Y1007+Y1008, Cat. No. Ab32101), p-STAT5 (Y694, Cat. No. Ab32364) were purchased from Abcam. JAK2 (Cat. No.2802) was obtained from Protein tech technology. p-STAT3 (Ser705, Cat. No. 340799), STAT3 (Cat. No. 200964), p-ERK (Thr202/204, Cat. No. 301245) and ERK (Cat. No. 201246-43F) were purchased from ZENBIO. STAT5 (Cat. No. 94205S) was obtained from Cell Signaling Technology. β-Actin (Cat. No. AB0035), Goat anti-mouse IgG (H+L) HPR (Cat. No. AB0102), and Goat anti-rabbit IgG (H+L) HPR (Cat. No. AB0101) antibodies were purchased from Abways technology.

## **Surface plasmon resonance (SPR) assay**

SPR assay was performed using Open SPRTM (Nicoya). Purified ligand proteins JAK2 JH1, JAK2 JH2 and JAK2 JH2V617F (both of them have His-tag) were immobilized to the surface of NTA sensor chip respectively. Then, the analyte FM was ﬂowed over the sensor chip in running buffer (10 mM HEPES, pH 7.4, 120 Mm NaCl, 3 mM EDTA, 0.005% (v/v) Tween20) at a ﬂow rate of 20μL/min. The chip surface was regenerated by 10 mM glycine–HCl, pH 2.0. The analysis software used in this experiment was TraceDrawer (Ridgeview Instruments ab, Sweden).

## Protein expression and purification

The kinase domain of human JAK2 JH2 (residues 536-832), wild-type (JAK2 JH2) and V617F (JH2V617F) were expressed and purified according to a previously described protocol [26]. Briefly, a codon-optimized gene encoding human JAK2 JH2 was subcloned into the pFastBac with a C-terminal thrombin-cleavable hexahistidine tag, then the protein JAK2 JH2 and JAK2 JH2V617F were overexpressed in baculovirus-infected Spodoptera frugiperda (Sf9) insect cells, which were grown at 27°C in SF Medium (Gibco) and harvested 72 hours after infection. Cells were resuspended in a lysis buffer containing 25 mM Tris-HCl (pH 8.0), 250 mM NaCl, 1 mM DTT and 20 mM imidazole, supplemented with complete protease inhibitors mixture (Roche Diagnostics). Cells were lysed by an Emulsiflex homogenizer, then centrifuged at 20000 g for 1 hour. The supernatant was filtered and incubated with Ni-NTA resins (GE-Healthcare) for nickel-affinity chromatography. Subsequently, the protein was assessed using anion-exchange chromatography (Source-Q, GE Healthcare) for further purification. Then, the protein was purified by gel filtration chromatography using Superdex 200 column (GE-Healthcare) equilibrated in 20 mM Tris pH 8.0, 100 mM NaCl and 1 mM DTT. The fractions of JAK2 JH2 were concentrated to 10 mg/mL, flash frozen in liquid nitrogen and stored at -80 °C for crystallization studies.

## **Crystallization** and crystal soaking

The crystals of JAK2 JH2 were grown at 16°C using the hanging drop vapor-diffusion method in a reservoir solution containing 100 mM Tris-HCl (pH 8.0), 200 mM sodium acetate and 18% (w/v) PEG4000. Diffraction quality crystals formed after 5-8 days. For crystal soaking, 0.2μL of FM (dissolved in DMSO at 10 mM concentration) was added to a 2μL crystal containing drop for 24 hours at 16 °C. The crystals were cryoprotected in reservoir solution supplemented with 20% glycerol (v/v) and flash frozen by liquid nitrogen prior to data collection.

## **Diffraction and structure determination**

X-ray diffraction data of the JAK2 JH2 in complex with FM were collected at BL19U1 beamline in Shanghai Synchrotron Radiation Facility (Shanghai, China). The collected data were indexed, integrated and scaled using HKL3000 [27]. The complex structure was determined by molecular replacement with the program PHASER [28] using the JAK2 JH2/JNJ-7706621 (PDB ID 5USZ) as the search model. The FM restraint was generated with eLBOW [29]. The final model was built in Coot [30] and refined in PHENIX [31]. The quality of the model were evaluated by MolProbity [32]. The structure figures were prepared using Pymol. The crystal structure of the JAK2 JH2/FM has been deposited in the Protein Data Bank with PDB ID codes 7F7W. See Table S6 for the data collection and refinement statistics.

## Production of retroviruses

293T cells were transfected with the retroviral vector MSCV-IRES-GFP carrying the *JAK2*^V617F^ cDNA. To detect the retrovirus titer, we incubated NIH-3T3 cells for 3 hours in DMEM supplemented with 10% fetal bovine serum, 8 µg/ml polybrene and 1M HEPES (Cat. No. XBPY, MACKLIN). The proportion of GFP-positive cells was measured to estimate retrovirus titer by flow cytometry.

## **Flow cytometry**

For cell cycle analysis, cells were incubated with compounds for 24 hours, and harvested the cells with PBS once. Cells were fixed with 75% ethanol and stored at 4 ℃ overnight. The next day, fixed cells were washed with ice-cold PBS and re-suspended in 500μL propidium iodide solution (50μg/mL propidium iodide, 0.1% NP-40, 0.1% sodium citrate, 0.1% Triton X-100), and incubated for 15 min at 37 °C. The flow cytometry results were analyzed with Modfit software for the proportion of the cell population.

For apoptosis assay, cells were incubated with compounds or DMSO for 48 hours, and following the manufacturer’s instructions of the apoptosis detection kit (Cat. No. 88-8807-72, Thermo Fisher Scientific). Cells were re-suspended in 100μL of binding buffer, and then, each group was stained with 5μL of Annexin V solution for 20 min. After washing the cells once with binding buffer and stained with 5μL of the PI solution, it was placed in the dark and analyze by flow cytometer within 1 h. FlowJo10 software was used to analyze the proportion of the cell population.

For Ba/F3-*EPOR*-*JAK2*^V671F^ disease models, cells from peripheral blood were treated with red blood cell lysate, and re-suspended in 500μL ice-cold PBS. The proportion of GFP-positive cells were analyzed and represented with Flowjo10.

For the *JAK2*^V617F^ BMT mouse model, cells from peripheral blood, spleen and bone marrow were treated with red blood cell lysate, and washed with PBS. Cells were incubated with various antibodies. Anti-mouse CD4 (Cat. No. 25-0041-82), anti-Hu/Mo CD45R (B220) (Cat. No. 17-0452-82), anti-Mo CD8a (Cat. No. 12-0081-82), anti-Mo LY-6G/Ly-6C (Cat. No. 17-5931-81), and anti-Mo CD11b (Cat. No. RM2804-3) were purchased from Invitrogen (Thermo Fisher Scientific, USA). Anti-mouse CD71 (Cat. No.113807) and anti-mouse TER-119 (Cat. No.116212) were obtained from Biolegend (USA). All stained cells were measured by flow cytometry (AttuneTMNxT, Acoustic Focusing Cytometer, Thermo Fisher Scientific, USA).

## Animal models

*Ba/F3-JAK2^V617F^ disease model*

3.0×10^6^ Ba/F3-*JAK2*^V671F^ cells were re-suspended in 100 μL ice-cold PBS and inoculated intravenously into female BALB/c nude mice. Mice in good condition and similar disease progression were randomized into four groups and treatment of vehicle, FM 15 and 30 mg/kg, and fedratinib 30mg/kg twice a day on day 4 after inoculation. When the weight of spleen in vehicle group exceeded 400 mg, mice were sacrificed. We also monitored the survival of mice every day, and killed moribund mice as the endpoint, and statistically analyze the survival. The inhibition of spleen growth was measured by spleen growth inhibition (%) = (spleen weight of vehicle group- spleen weight of treat group) / (spleen weight of vehicle group- spleen weight of normal group) ×100%.

*EPOR-JAK2^V617F^-driven murine malignancy model*

1.0×10^6^ Ba/F3-*EPOR*-*JAK2*^V671F^ cells were intravenously into female BALB/c nude mice, compounds were given by oral gavage bid. on the next day after cell inoculation. Similarly, we monitored the survival of mice every day, and the mice were sacrificed after 16-day treatment. Hematological parameters were measured by the National Chengdu Center for Safety Evaluation of Drugs (NCCSED).

*JAK2^V617F^-induced bone marrow transplantation (BMT) model*

BMT mouse model were established as previously described [33]. 200mg/kg 5-fluorouracil (Cat. No. 51-21-8, Sigma) was intravenously injected into male BALB/c donor mice. Four days later, bone marrow cells were harvested from the femur and tibia and cultured for 24 hours in DMEM supplemented with 10% FBS, penicillin/streptomycin, L-glutamine (Cat.No.XBPY, MACKLIN), 1M HEPES, ciprofloxacin(Cat. No. 93107-08-5, BBI Life Science), 10 µg/ml IL-3(Cat. No. 213-13), 10µg/ml IL-6(Cat. No. 216-16), 50 µg/ml stem cell factor (Cat. No. 250-03) (Peprotech, Rocky Hill, NJ) and 3µg/ml polybrene. The cells were subsequently infected with retroviral supernatants (MSCV-IRES-*JAK2*^V617F^-GFP) containing 8 µg/ml polybrene and 1M HEPES for 3 hours, and the cells were treated with retroviral supernatants again the next day. At the same time, BALB/c recipient mice were lethally irradiated with 5Gy, and then 1.0×10^6^ donor cells were intravenously injected into the lateral tail vein. Tumor implantation of mice was analyzed by FACS as described previously.

## PK and PD studies

We established the Ba/F3-*EPOR*-*JAK2*^V617F^ disease model to explore pharmacokinetics (PK) and pharmacodynamics (PD) studies in single and repeated doses of FM at 30 mg/kg dose. In single dose study, mice were randomly collected and analyzed by PB of flow cytometry on the 9^th^ day after inoculation. Mice were sacrificed at 0, 0.25, 0.5, 1, 4, 8, 12, and 24 hours after a single oral administration, and tissue samples were harvested to determine the distribution of FM by UFLC-MS/MS. UFLC system: SIL-30AC auto-sampler, LC-30AD chromatograph CBM-20A communications bus module， CTO-20AC prominence column oven, Shimadzu, Japan; MS system: Qtrap 5500, American AB Company.

For Ba/F3-*EPOR*-*JAK2*^V617F^ driven malignant model after repeated administration, tumor-bearing mice were randomly divided into 8 groups on the 10^th^ day. Mice were sacrificed at 0, 0.25, 0.5, 1, 4, 8, 12, and 24 hours after the last oral administration, and tissue samples were harvested to determine the distribution of FM and PK parameters by UFLC-MS/MS. For PD studies, we weighed the spleen, and analyzed the ratio of GFP^+^ in the spleen and PB by flow cytometry, and test the downstream pathway protein p-STAT5 (Y694, Cat. No. Ab32364) via Western Blot.

## Histologic analysis

Spleens, livers and bone marrows were harvested from the tumor bearing mice and fixed with 4% paraformaldehyde (Cat. No. G1101, Servicenio). All samples were embedded in paraffin and made into 5μm thick continuous sections. H&E, Tunel, Ki67, IHC and fibrosis staining were performed.

## Statistical analysis

Statistical analysis was analyzed by GraphPad Prism 7.0. Comparison between two groups was performed with two-sided Student *t*-test, and P < 0.05 was considered statistically significant. Survival data was evaluated by log-rank (Mantel-Cox) test.

# Supplementary Results

## FM could simultaneously bind with JH1 and JH2 of JAK2

The RMSD values for the JAK2 JH1 and JAK2 JH2 backbone atoms and non-hydrogen atoms of FM were calculated according to the structures obtained by docking and have been illustrated (Fig. S1A). The binding model for FM with JH1 and JH2 were shown in the Fig. S1B. Small fluctuations indicated that the entire system was stabilized. The snapshots obtained at 20 ns, 40 ns, 60 ns, 80 ns, and 100 ns from the MD trajectory and have been depicted for the two complex systems (Fig. S1E-F). The ligand remained securely in the pocket. Only the hydroxyl on the ligand showed fluctuations in the simulation time. Therefore, the JAK2 JH1 and JAK2 JH2 are stable when FM located at the active binding site. The hydrogen bonds that formed between FM and JAK2 were then examined. Hydrogen bond number and occupancy analyses over the MD trajectories are summarized (Fig. S1C and Table S3). There are two stable hydrogen bonds formation between FM and the hinge loop of JAK2 JH1 and JAK2 JH2 which occupancy is more than 95%. The third hydrogen bond for JAK2-JH2 is unstable in the simulation. Therefore, we can conclude that the hydrogen bond mainly contributed to the FM binding with JAK2 JH1 and JAK2 JH2. Here, we also used MM/GBSA to calculate the absolute binding free energies for FM with JAK2 JH1 and FM with JAK2 JH2 systems (Fig. S1D and Tables S4-5). The $\Delta G_{\mathrm{bind}}^{\mathrm{cal}}$ of FM/JAK2 is around -16.06 and -20.26kcal/mol for JH1 and JH2, respectively (Fig. S1D). We could speculate that FM can simultaneously bind with JH1 and JH2 of JAK2. Overall, the calculated entropies and enthalpies can suggest that the formation of binding complexes is an enthalpy driven process. Meanwhile, it has been accepted that the binding of FM with JAK2 is mainly due to nonpolar interactions. In other words, the hydrophobic interaction (mainly from van der Waals interactions) is the dominant factor in the formation of binding system.

## PK/PD of FM in Ba/F3-*EPOR*-*JAK2*^V617F^ driven mouse model

FM had been proved to have a significant therapeutic effect on Ba/F3-*EPOR*-*JAK2*^V617F^ driven MPN model. To further study the pharmacokinetic (PK) and pharmacodynamic (PD) characteristics of FM *in vivo*, we used UFLC-MS/MS method to determine the distribution and PK characteristics of FM in Ba/F3-*EPOR*-*JAK2*^V617F^ tumor-bearing mice. On the 10^th^ day after inoculation, tumor cell proportions in PB, spleen, and bone marrow of tumor-bearing mice were 15.0 ± 4.4%, 27.1 ± 9.2%, and 21.7 ± 3.2%, respectively (Fig. S4A). Then, mice were randomly divided into nine group (n = 6) and sacrificed at 0, 0.25, 0.5, 1, 2, 4, 8, 12, and 24 h after FM 30 mg/kg single-dose oral administration for tissue distribution and PK analysis. In addition to stomach and intestine, FM was mainly distributed in liver, spleen, lung, and kidney, while rarely distributed in brain and skeletal muscle, indicating that FM had low neurotoxicity (Fig. S4B-C, Tables S8-9a). After 8 hours of administration, the concentration of FM in the spleen was 1410.80 ± 967.58 ng/g. By calculation, the drug concentration in the spleen reached 2416 ± 1657 nmol/kg (approximately 2416 nmol/L), which was significantly higher than the IC_50_ value of Ba/ F3-*EPOR*-*JAK2*^V617F^ cells *in vitro* at 790 ± 10 nmol/L. After 12 hours administration, the concentration of FM in spleen was 358.48 ± 228.18 ng/g, approximately 614 ± 391 nmol /L, close to the IC_50_ value of Ba/F3-*EPOR*-*JAK2*^V617F^ cells *in vitro* (Table S8). These results indicated that 0.25-12 hours after administration, the concentration of FM in spleen was greater than or close to the IC_50_ value of the cells *in vitro*, which could significantly inhibit proliferation and induce apoptosis of tumor cells, further demonstrating the rationality and feasibility of twice-daily administration of FM.

In tumor-bearing mice, the exposure AUC_(0-24h)_ in plasma and spleen were 2990.15 ± 557.79 g/L*h and 45706.71 ± 9606.67 g/L*h, respectively, and the C_max_ was 1028.18 ± 173.54 ng/mL and 12009.71 ± 3473.35 ng/g, respectively. The AUC_(0-24h)_ and C_max_ of spleen were 15.29 times and 11.68 folds higher than that of plasma, indicating that FM was eliminated rapidly in plasma, and had a certain accumulation in spleen, which contributed to improved antitumor ability and reduced toxicity.

Then, we established the Ba/F3-*EPOR*-*JAK2*^V617F^ mouse model to further study the PK/PD characteristics of FM 30 mg/kg long-term administration. After 10 days of modeling, the GFP ^+^ tumor cells in PB and spleen of untreated mice were 29.18 ± 10.35% and 26.14 ± 9.18%, respectively (Fig. S4D). Consistent with the single administration, FM was mainly distributed in liver, spleen, lung, and kidney, but rarely in brain and skeletal muscle (Table S9b). The plasma and tissue drug concentrations of tumor-bearing mice after repeated administration was shown in Fig.5 A-B and Tables S8 and S9b. 8 hours and 12 hours after the last administration, the concentration of FM in spleen were 2151.00 ± 1385.33ng/g and 390.80 ± 181.44ng/g, which were greater than the IC_50_ *in vitro*, respectively. The exposure AUC_(0-24h)_ and C_max_ of spleen were 17.95 and 14.14 folds higher than that of plasma, respectively.

In addition, the PD study of FM was related to p-STAT5, and the results showed that FM significantly downregulated JAK signaling pathway (data not shown), and inhibited tumor progression. 8 hours after administration, the p-STAT5 level reached the lowest level, which was down-regulated by 43.80 ± 1.59%. With the decrease of drug concentration, the level of p-STAT5 increased to a certain extent, but 12 hours after administration, the level of p-STAT5 still decreased (Fig. 5C). Before the last administration, the GFP ^+^ tumor cells in PB and spleen were 19.75 ± 12.93% and 17.12 ± 8.15%, respectively, and 12 hours after the 17th administration, the GFP ^+^ tumor cells in PB and spleen were decreased at 11.77 ± 5.5% and 11. 71 ± 7. 91%, respectively. In addition, the weight of spleen was 267.2 ± 56.8 mg in the untreated group and 167.5 ± 52.9 mg in the repeated administration groups (12 hours after the last administration) (Fig. 5D and Fig. S4E). Furthermore, FM exhibited significant anti-tumor effect in PB and spleen of Ba/F3-*EPOR*-*JAK2*^V617F^ tumor-bearing mice (Fig. 5E and Fig. S4F). H&E and Tunel staining showed that FM inhibited tumor invasion and considerably induced tumor cell apoptosis. Ki67 and IHC staining exhibited that the number of tumor cells in a cell proliferation state decreased and the downstream pathway of p-STAT3 was inhibited (Fig. 5F). At 12 h treatment, IHC analysis from BM showed that the protein levels of p-STAT3 and p-STAT5 were significantly down-regulated (Fig. 5G).

## Myelosuppresive effects of FM on CD34^+^ cells

To further understand the effect of FM on normal mice and normal human CD34^+^ cells, we conducted a further analysis on CD34^+^ cells. For normal mice, we measured the proportion of CD34^+^ in the peripheral blood of BALB/c mice on Day 0, and then mice were treated with FM 30 mg/kg p.o. twice a day. As shown in Fig. S6A, the CD34^+^ cells were significantly inhibited after FM treatment. To investigate the effect of FM on normal human CD34^+^ cells, we collected mononuclear cells of PB acquired from healthy donors. Mononuclear cells were treated with different concentrations of FM for 48 h, and FM showed a general inhibitory effect on normal human CD34^+^ cells (Fig. S6B). Overall, these data indicated that FM has potential myelosuppressive effects. Therefore, in the future phase I clinical trials, we should examine the myelosuppressive effects of FM in patients with MPN.

**References**

1. Schrödinger L. The PyMOL Molecular Graphics System. (2010).

2. Sanner MF. Python: A programming language for software integration and development. *J Mol Graph*. 17(1), 57-61 (1999).

3. Stewart JJP. Optimization of parameters for semi-empirical methods I-method. *J Comput Chem*. 10(2), 209-220 (1989).

4. Stewart JJP. MOPAC: A semiempirical molecular orbital program. *J Comput-Aided Mol Des*. 4(1), 1-45 (1990).

5. Gasteiger J, Marsili M. Iterative partial equalization of orbital electronegativity—a rapid access to atomic charges. *Tetrahedron*. 36(22), 3219-3228 (1980).

6. Morris GM, et al. AutoDock4 and AutoDockTools4: Automated Docking with Selective Receptor Flexibility. *J Comput Chem*. 30(16), 2785-2791 (2009).

7. Tian C, et al. ff19SB: Amino-Acid-Specific Protein Backbone Parameters Trained against Quantum Mechanics Energy Surfaces in Solution. *Journal of Chemical Theory and Computation*. 16(1), 528-552 (2020).

8. Wang JM, Wolf RM, Caldwell JW, Kollman PA, Case DA. Development and testing of a general amber force field. *J Comput Chem*. 25(9), 1157-1174 (2004).

9. Frisch MJ, et al. Gaussian 09. Wallingford, CT, USA: Gaussian, Inc.; 2009.

10. Bayly CI, Cieplak P, Cornell WD, Kollman PA. A WELL-BEHAVED ELECTROSTATIC POTENTIAL BASED METHOD USING CHARGE RESTRAINTS FOR DERIVING ATOMIC CHARGES - THE RESP MODEL. *J Phys Chem*. 97(40), 10269-10280 (1993).

11. Jorgensen WL, Chandrasekhar J, Madura JD, Impey RW, Klein ML. Comparison of simple potential functions for simulating liquid water. *The Journal of Chemical Physics*. 79(2), 926-935 (1983).

12. Darden T, York D, Pedersen L. Particle mesh Ewald: An N⋅log(N) method for Ewald sums in large systems. *The Journal of Chemical Physics*. 98(12), 10089-10092 (1993).

13. Ryckaert J-P, Ciccotti G, Berendsen HJC. Numerical integration of the cartesian equations of motion of a system with constraints: molecular dynamics of n-alkanes. *Journal of Computational Physics*. 23(3), 327-341 (1977).

14. Pan W, et al. The cytochrome c-cyclo 6 aramide complex as a supramolecular catalyst in methanol. *New J Chem*. 42(5), 3857-3866 (2018).

15. Wang XQ, et al. Purification and biochemical characterization of FrsA protein from Vibrio vulnificus as an esterase. *PLoS One*. 14(4), 13 (2019).

16. Cheatham T, Galindo R, Roe D. Parallel analysis of large ensembles of molecular dynamics simulation derived trajectories with the open-source CPPTRAJ tools. *Abstracts of Papers of the American Chemical Society*. 257, 1 (2019).

17. Roe DR, Cheatham TE. Parallelization of CPPTRAJ Enables Large Scale Analysis of Molecular Dynamics Trajectory Data. *J Comput Chem*. 39(25), 2110-2117 (2018).

18. Roe DR, Cheatham TE. PTRAJ and CPPTRAJ: Software for Processing and Analysis of Molecular Dynamics Trajectory Data. *Journal of Chemical Theory and Computation*. 9(7), 3084-3095 (2013).

19. Srinivasan J, Cheatham TE, Cieplak P, Kollman PA, Case DA. Continuum solvent studies of the stability of DNA, RNA, and phosphoramidate - DNA helices. *J Am Chem Soc*. 120(37), 9401-9409 (1998).

20. Miller BR, McGee TD, Swails JM, Homeyer N, Gohlke H, Roitberg AE. MMPBSA.py: An efficient program for end-state free energy calculations. *J Chem Theory Comput*. 8(9), 3314-3321 (2012).

21. Weiser J, Shenkin PS, Still WC. Approximate atomic surfaces from linear combinations of pairwise overlaps (LCPO). *J Comput Chem*. 20(2), 217-230 (1999).

22. Srinivasan J, Trevathan MW, Beroza P, Case DA. Application of a pairwise generalized Born model to proteins and nucleic acids: inclusion of salt effects. *Theor Chem Acc*. 101(6), 426-434 (1999).

23. Still WC, Tempczyk A, Hawley RC, Hendrickson T. Semianalytical treatment of solvation for molecular mechanics and dynamics. *J Am Chem Soc*. 112(16), 6127-6129 (1990).

24. Onufriev A, Bashford D, Case DA. Exploring protein native states and large-scale conformational changes with a modified generalized born model. *Proteins*. 55(2), 383-394 (2004).

25. Case DA. Normal mode analysis of protein dynamics. *Curr Opin Struct Biol*. 4(2), 285-290 (1994).

26. Bandaranayake RM, Ungureanu D, Shan Y, Shaw DE, Silvennoinen O, Hubbard SR. Crystal structures of the JAK2 pseudokinase domain and the pathogenic mutant V617F. *Nat Struct Mol Biol*. 19(8), 754-759 (2012).

27. Minor W, Cymborowski M, Otwinowski Z, Chruszcz M. HKL-3000: the integration of data reduction and structure solution--from diffraction images to an initial model in minutes. *Acta Crystallogr D Biol Crystallogr*. 62(Pt 8), 859-866 (2006).

28. McCoy AJ, Grosse-Kunstleve RW, Adams PD, Winn MD, Storoni LC, Read RJ. Phaser crystallographic software. *J Appl Crystallogr*. 40(Pt 4), 658-674 (2007).

29. Moriarty NW, Grosse-Kunstleve RW, Adams PD. electronic Ligand Builder and Optimization Workbench (eLBOW): a tool for ligand coordinate and restraint generation. *Acta Crystallogr D Biol Crystallogr*. 65(Pt 10), 1074-1080 (2009).

30. Emsley P, Lohkamp B, Scott WG, Cowtan K. Features and development of Coot. *Acta Crystallogr D Biol Crystallogr*. 66(Pt 4), 486-501 (2010).

31. Adams PD, et al. PHENIX: a comprehensive Python-based system for macromolecular structure solution. *Acta Crystallogr D Biol Crystallogr*. 66(Pt 2), 213-221 (2010).

32. Chen VB, et al. MolProbity: all-atom structure validation for macromolecular crystallography. *Acta Crystallogr D Biol Crystallogr*. 66(Pt 1), 12-21 (2010).

33. Pardanani A, Lasho T, Smith G, Burns CJ, Fantino E, Tefferi A. CYT387, a selective JAK1/JAK2 inhibitor: in vitro assessment of kinase selectivity and preclinical studies using cell lines and primary cells from polycythemia vera patients. *Leukemia*. 23(8), 1441-1445 (2009).

# Supplementary **Figure legends**

## Figure S1. FM could bind to JH1 and JH2 through molecular docking study.

(A) The mean square root deviation (RMSD) value of heavy atoms of JAK2 protein and FM among 100 ns MD simulations. (B) Interaction between FM/JAK2-JH1 and FM/JAK2-JH2 complex system, for the representative conformation of the largest cluster from the cluster analysis. (C) Hydrogen bond number analysis of interactions between FM/JAK2-JH1 and FM/JAK2-JH2. Occupancy was expressed as % of the period (100 ns) during which specific hydrogen bonds were formed. Hydrogen bond was defined as the distance between the acceptor and donor atoms < 3.5 Å, with an internal angle between the H-acceptor and H-donor > 120º. (D) Binding free energies ($\Delta G_{\mathrm{bind}}^{\mathrm{cal}}$) for FM/JAK2-JH1 and FM/JAK2-JH2 complexes and decomposition to electrostatic interaction ($E_{\mathrm{ele}}$), van der Walls interaction ($E_{\mathrm{vdW}}$), solvation free energies ($E_{\mathrm{GB}}$), and entropy ($TS_{\mathrm{total}}$). Energy values were presented in kcal/mol. (E) Snapshots of the FM/JAK2-JH1 along the dynamic simulation time for 20, 40, 60, 80 and 100 ns. For clarity, the water molecules have been removed. The FM is plotted using stick style, while cartoon style for JAK2 JH1. (F) Snapshots of the FM/JAK2-JH2 along the dynamic simulation time for 20, 40, 60, 80 and 100 ns. For clarity, the water molecules have been removed. The FM is plotted using stick style, while cartoon style for JAK2 JH2.

## Figure S2. FM exhibits potent activity *in vitro*.

(A) Inhibition of cytokine induced JAK auto-phosphorylation by FM in HEL cell lines. Raw data about HEL (B) and Ba/F3-*JAK2*^V617F^ (C) cells were supplemented with different concentrations of FM for cell cycle analysis. Flow cytometry analysis of apoptosis in HEL (D) and MV-4-11 (E) cells after 48h treatment of FM, ruxolitinib and fedratinib at the indicated concentrations. Data are represented as mean ± SD, *p < 0.05, **p < 0.01, ***p < 0.001 *vs*. FM treatment groups at the same dose, *t*-test. (F) p-FLT3, p-STAT5, and p-ERK in Molm-13 cell line were also assessed after 3 hours exposure with increasing concentrations of FM.

## Figure S3. Effects of FM in MPN mouse model.

(A) In Ba/F3-*JAK2*^V617F^ bearing mice, body weight was analyzed (n = 6). (B) In Ba/F3-*EPOR*-*JAK2*^V617F^ mouse model, representative images showing H&E staining sections of heart, lung, and kidney, and p-JAK2 levels were analyzed via IHC in spleen, magnification ×200. The histogram on the right panel is the quantitative statistics of IHC staining results performed by Image-Pro Plus. Data are represented as mean ± SD, *p < 0.05, **p < 0.01, ***p < 0.001 *vs*. vehicle, *t*-test.

## Figure S4. Pharmacokinetics (PK) and pharmacodynamics (PD) of FM in the Ba/F3-*EPOR*-*JAK2*^V617F^ disease model.

In single oral administration of FM 30mg/kg mouse model, tumor-bearing mice were randomly divided into 8 groups and sacrificed at different time points (0-24 h) after administration. (A) 10 days after inoculation, tumor burden in BM, spleen, and PB were monitored by FACS (n = 6). (B-C) FM concentration in different organizations after a single FM 30 mg/kg by oral gavage at different time points (n = 6). In repeated oral administration of FM 30mg/kg mouse model, (D) mice were analyzed on day 9 after inoculation, and tumor burden was detected in non-/repeated -administration groups of tumor-bearing mice (n = 6). (E) Percentages of GFP^+^ cells in PB, spleen, and BM were analyzed after 1×10^6^ Ba/F3-*EPOR*-*JAK2*^V617F^ cells inoculation (n = 3). (F) Mice were sacrificed at the end of the administration and the spleen size was photographed (n = 6). Data were represented as mean ± SD.

## Figure S5. FM showed anti-tumor effects in the *JAK2*^V617F^ bone marrow transplantation (BMT) mouse model of myelofibrosis *in vivo*.

（A）GFP^+^ cells in PB reached 13.53 ± 2.64% (baseline burden). Mice were divided into 5 groups and then treated with vehicle, FM (15 and 30 mg/kg) and Fedratinib 30 mg/kg orally twice daily. (B) PB were analyzed for the number of neutrophils and lymphocytes during 112 days. (C) 14 days after BMT, mice were randomized to receive treatment. FACS analysis showed the percentage of GFP^+^Gr1^+^ cells in PB for the vehicle or FM treatment groups of *JAK2*^V617F^ mutation BMT bearing mice at different time points. (D) FACS analyzed the percentage of Gr1^+^(Granulocytes), Mac1^+^(Macrophages), CD71^+^(early erythroid progenitors) and Ter119^+^(late erythroid progenitors) in GFP^+^ cells in BM. Data are represented as mean ± SD, *p < 0.05, **p < 0.01, ***p < 0.001 *vs.* vehicle, *t*-test.

## Figure S6. FM showed potential myelosuppresive effects on normal mice and normal human CD34^+^ cells.

(A) Normal BALB/c mice were treated with vehicle and FM 30mg/kg p.o. twice a day. FACS analysis of the percentage of CD34^+^ cells on Day 0, 7, 14 and 21 (n = 7). (B) Mononuclear cells were isolated from the PB of healthy donors and CD34^+^ cells were analyzed after incubated with FM after 48h. Data are represented as mean ± SD, *p < 0.05, **p < 0.01, ***p < 0.001 *vs.* vehicle, *t*-test.
