# Supplementary material for: Preclinical studies of Flonoltinib Maleate, a novel JAK2/FLT3 inhibitor, in treatment of JAK2V617F-induced myeloproliferative neoplasms
Source: Blood Cancer J. 2022 Mar 7;12(3):37. doi: 10.1038/s41408-022-00628-2 (PMC8901636; doi:10.1038/s41408-022-00628-2)
Supplement: Supplementary file 2 — Supplementary Table [file 41408_2022_628_MOESM2_ESM.docx]

# **Table S1.** Clinical and genetic characteristics of MPN patients.

| **Sample ID** | **Age**  **(y)** | **Sex** | **Diagnosis** | **JAK2** | **CALR** | **MPL** | **WBC**  **(10^9^/L)** | **HGB (g/dL)** | **HCT**  **(%)** | **PLT (10^9^/L)** | **Splenomegaly** |
| --- | --- | --- | --- | --- | --- | --- | --- | --- | --- | --- | --- |
| 1# | 49 | F | ET | ＋ | － | NA | 11.00 | 138 | 0.41 | 1025 | No |
| 2# | 51 | F | PV | ＋ | － | NA | 14.42 | 197 | 0.60 | 834 | Yes |
| 3# | 61 | M | ET | ＋ | － | NA | 8.97 | 167 | 0.49 | 643 | No |
| 4# | 51 | F | ET | ＋ | － | NA | 5.97 | 126 | 0.46 | 743 | Yes |
| 5# | 53 | F | PV→MF | ＋ | － | NA | 10.89 | 167 | 0.58 | 204 | Yes |

Abbreviations: WBC, white blood cells; Hg B, hemoglobin B; HCT, hematocrit; NA, not available.

# **Table S2.** *In vitro* enzyme inhibition activity of FM compared to other JAK2 inhibitors.

| Enzyme IC_50_ (nM) | JAK2 Inhibitors | | | | |
| --- | --- | --- | --- | --- | --- |
|  | FM | Ruxolitinib[11] | Fedratinib[12] | Pacritinib[13] | Momelotinib[8] |
| JAK1 | 690 | 3.3 | 105 | 1280 | 11 |
| JAK2 | 0.8 | 2.8 | 3 | 23 | 18 |
| JAK2V617F | 1.4 | NA | 3 | 19 | NA |
| JAK3 | 557 | 428 | 1002 | 520 | 155 |
| TYK2 | 65 | 19 | 405 | 50 | 17 |
| FLT3 | 15 | NA | 15 | 22 | NA |

NA: not available;

Note: *In vitro* activity data of other JAK2 inhibitors are reported in the literature.

**Table S3. Hydrogen bond network analysis of interactions between FM and JAK2 JH1 and JAK2 JH2.**

| Hydrogen Bond | Occupancy | Average Distance | Average Angle |
| --- | --- | --- | --- |
| FM@N1_JH2: VAL629@N | 98.12 | 3.05 | 152.52 |
| FM@N3_JH2: VAL629@O | 96.76 | 3.03 | 157.80 |
| FM@N6_JH2: LYS581@NZ | 57.60 | 3.11 | 141.35 |
| FM@N3_JH1: LEU932@O | 98.64 | 2.97 | 153.59 |
| FM@N1_JH1: LEU932@N | 97.52 | 3.14 | 160.82 |

Occupancy is expressed as % of the period (100 ns) during which specific hydrogen bonds are formed. Hydrogen bond is defined as the distance between the acceptor and donor atoms < 3.5 Å, with an internal angle between the H-acceptor and H-donor > 120º.

**Table S4. Binding free energies (**$\boldsymbol{\Delta}\mathbf{G}_{\mathbf{bind}}^{\mathbf{cal}}$**) for FM/JAK2-JH1 complexes and decomposition to electrostatic interaction (**$\mathbf{E}_{\mathbf{ele}}$**), van der Walls interaction (**$\mathbf{E}_{\mathbf{vdW}}$**), solvation free energies (**$\mathbf{E}_{\mathbf{GB}}$**), and entropy (**$\mathbf{T}\mathbf{S}_{\mathbf{total}}$**).**

|  | Complex | Receptor | Ligand | Differences |
| --- | --- | --- | --- | --- |
| $E_{\mathrm{vdW}}$ | -2247.41(20.96) | -2201.07(20.75) | -3.83(1.82) | -42.50(2.94) |
| $E_{\mathrm{ele}}$ | -20017.03(117.73) | -19985.16(117.41) | -14.29(5.73) | -17.58(3.68) |
| $E_{\mathrm{GB}}$ | -3446.44(93.73) | -3457.13(93.78) | -17.12(0.88) | 27.82(3.18) |
| $E_{\mathrm{surf}}$ | 96.90(1.76) | 97.51(1.74) | 4.53(0.05) | -5.14(0.30) |
| $G_{\mathrm{gas}}$ | -4823.29(109.87) | -4750.72(109.94) | -12.48(6.14) | -60.08(4.72) |
| $G_{\mathrm{solv}}$ | -3349.53(92.88) | -3359.62(92.92) | -12.59(0.86) | 22.68(3.06) |
| $E_{\mathrm{gas}}+G_{\mathrm{sol}}$ | -8172.82(48.73) | -8110.34(48.54) | -25.07(5.97) | -37.41(3.37) |
| $TS_{\mathrm{total}}$ | 3102.41(8.74) | 3058.80(9.04) | 64.95(0.21) | -21.34(5.80) |
| $\Delta G_{\mathrm{bind}}^{\mathrm{cal}}$ |  |  |  | -16.06(6.71) |

Energy values are presented in kcal/mol. Uncertainties shown in parentheses were calculated as the root mean square error for all frames extracted from the trajectories.

**Table S5. Binding free energies (**$\boldsymbol{\Delta}\mathbf{G}_{\mathbf{bind}}^{\mathbf{cal}}$**) for FM/JAK2-JH2 complexes and decomposition to electrostatic interaction (**$\mathbf{E}_{\mathbf{ele}}$**), van der Walls interaction (**$\mathbf{E}_{\mathbf{vdW}}$**), solvation free energies (**$\mathbf{E}_{\mathbf{GB}}$**), and entropy (**$\mathbf{T}\mathbf{S}_{\mathbf{total}}$**).**

|  | Complex | Receptor | Ligand | Differences |
| --- | --- | --- | --- | --- |
| $E_{\mathrm{vdW}}$ | -2457.64(26.55) | -2405.47(26.33) | -3.76(1.80) | -48.41(4.58) |
| $E_{\mathrm{ele}}$ | -21448.85(134.08) | -21420.76(134.17) | -12.51(5.66) | -15.58(4.61) |
| $E_{\mathrm{GB}}$ | -3288.77(118.22) | -3300.09(117.65) | -17.16(0.87) | 28.47(5.05) |
| $E_{\mathrm{surf}}$ | 102.92(2.53) | 103.98(2.54) | 4.51(0.06) | -5.57(0.58) |
| $G_{\mathrm{gas}}$ | -6889.61(139.97) | -6814.27(139.87) | -11.35(5.92) | -63.99(7.24) |
| $G_{\mathrm{solv}}$ | -3185.85(116.66) | -3196.11(116.03) | -12.65(0.85) | 22.91(4.66) |
| $E_{\mathrm{gas}}+G_{\mathrm{sol}}$ | -10075.46(56.08) | -10010.38(55.87) | -24.00(5.80) | -41.08(3.88) |
| $TS_{\mathrm{total}}$ | 3374.50(10.16) | 3330.24(10.89) | 65.08(0.26) | -20.82(7.25) |
| $\Delta G_{\mathrm{bind}}^{\mathrm{cal}}$ |  |  |  | -20.26(8.22） |

Energy values are presented in kcal/mol. Uncertainties shown in parentheses were calculated as the root mean square error for all frames extracted from the trajectories.

## **Table S6. Data collection and refinement statistics.**

| Protein/Small Molecule | JAK2-JH2/FM |
| --- | --- |
| Data Collection |  |
| Space Group | P 1 2_1_ 1 |
| *a, b, c* (Å) | 61.01, 57.80, 86.28 |
| α, β, γ (°) | 90.00, 109.32, 90.00 |
| Resolution (Å) * | 81.43-1.88(1.91-1.88) |
| Rsym or Rmeas (%)* | 7.2(82.8) |
| *I/σI** | 14.5(1) |
| Completeness (%)* | 94.4(30.3) |
| Redundancy* | 6.4(1.68) |
| Refinement |  |
| Resolution (Å) | 57.6 – 1.88 |
| Rwork/Rfree | 0.2068/0.2383 |
| Number of Atoms |  |
| Protein, Solvent, Ion/Small Molecule | 4457/237/68 |
| B Factors (Å2) | 29/50.8/45 |
| RMS Deviations |  |
| Bond (Å) | 0.007 |
| Angle (°) | 1 |

*Values in parentheses refer to the outer resolution shell.
Each data set was collected from a single crystal.

# Table S7. ***In vitro* activity of FM, Ruxolitinib and Fedratinib** against different hematological tumor cell lines.

| Cell line | Tumor type | Transforming kinase | IC_50_(μM) ± SD^a^ | | |
| --- | --- | --- | --- | --- | --- |
|  |  |  | FM | Ruxolitinib | Fedratinib |
| Ba/F3-*JAK2*^WT^ | Murine pre-B cell | IL-3–dependent growth | 0.39±0.20 | 0.19±0.01 | 0.68±0.12 |
| Ba/F3-*EPOR* | Murine pre-B cell | IL-3–dependent growth | 0.55±0.13 | 0.33±0.04 | 0.97±0.23 |
| HEL | Acute myeloid leukemia | JAK2V617F | 0.26±0.20 | 1.30±0.89 | 0.77±0.36 |
| Ba/F3-*JAK2*^V617F^ | Murine pre-B cell | JAK2V617F | 0.20±0.01 | 0.33±0.13 | 0.61±0.13 |
| Ba/F3-*EPOR*-*JAK2*^V617F^ | Murine pre-B cell | JAK2V617F | 0.78±0.21 | 0.87±0.24 | 1.71±0.04 |
| MV-4-11 | Acute myeloid leukemia | FLT3-ITD expression | 0.01±0.01 | >10 | 0.26±0.11 |
| Molm-13 | Acute myeloid leukemia | FLT3-ITD expression | 0.06±0.03 | >10 | 0.17±0.01 |
| Ba/F3-*FLT3*-*ITD* | Murine pre-B cell | FLT3-ITD expression | 0.12±0.01 | >10 | 0.21±0.05 |
| Ba/F3-*FLT3*-*ITD*^F691^ | Murine pre-B cell | FLT3-ITD expression | 0.15±0.02 | >10 | 0.74±0.15 |
| Ba/F3-*FLT3*-*ITD*^D835V^ | Murine pre-B cell | FLT3-ITD expression | 0.10±0.06 | >10 | 0.37±0.04 |

Abbreviations: FLT3-*ITD*, Fms-like tyrosine kinase 3–insert tandem duplication.

^a^ IC_50_ = compounds concentration required to inhibit tumor cell proliferation by 50%; data are expressed as the mean ± SD from the dose-response curves of at least three independent experiments

# Table S8. FM concentration in plasma and spleen of Ba/F3-*EPOR*-*JAK2*^V617F^ bearing mice after single or repeated oral administration (Mean ± SD; n = 6).

| Time-point (h) | Single administration | | Repeated administration | |
| --- | --- | --- | --- | --- |
|  | Plasma (ng/mL) | Spleen (ng/g) | Plasma(ng/mL) | Spleen (ng/g) |
| 0 | NA | NA | 5.60±1.53 | 68.98±44.84 |
| 0.25 | 652.94±108.28 | 5628.25±710.26 | 627.99±143.50 | 3702.90±1442.01 |
| 0.5 | 857.23±74.50 | 10416.00±2658.42 | 889.02±237.54 | 12069.43±1908.55 |
| 1 | 921.81±301.86 | 9928.81±3965.67 | 1042.04±375.10 | 17133.25±6423.90 |
| 2 | 457.59±88.18 | 8531.55±1505.63 | 637.74±168.47 | 12218.63±4390.76 |
| 4 | 247.96±80.80 | 3814.00±1876.53 | 292.37±100.53 | 6183.70±1679.40 |
| 8 | 59.92±49.99 | 1410.80±967.58 | 105.90±84.09 | 2151.00±1385.33 |
| 12 | 61.44±64.28 | 358.48±228.18 | 19.33±12.90 | 390.80±181.44 |
| 24 | NA | 32.65±13.27 | 5.56±4.97 | 46.79±21.67 |

NA: not available.

# Table S9a. FM concentration in tissues of Ba/F3-*EPOR*-*JAK2*^V617F^ bearing mice after a single oral administration­­­­ (Mean ± SD; n = 6).

| Time-point (h) | Tissues (ng/g) | | | | | | | |
| --- | --- | --- | --- | --- | --- | --- | --- | --- |
|  | Brain | Heart | Liver | Lung | Kidney | Stomach | Intestine | Muscle |
| 0 | NA | NA | NA | NA | NA | NA | NA | NA |
| 0.5 | 353.71±77.07 | 3385.17±183.90 | 12835.76±3699.01 | 11642.24±2960.72 | 12145.54±1255.81 | 30298.33±18393.93 | 14902.40±11448.21 | 633.05±618.93 |
| 2 | 308.82±40.52 | 1645.34±381.11 | 6533.39±2917.20 | 3823.76±739.14 | 6215.68±854.82 | 30453.60±29028.42 | 6250.34±3226.25 | 217.29±116.29 |
| 4 | 157.03±58.34 | 863.77±418.15 | 3821.26±1483.65 | 2085.26±512.99 | 2412.75±1014.52 | 17679.56±10912.19 | 8489.82±2243.06 | 151.97±73.88 |
| 8 | 38.36±28.48 | 199.43±145.02 | 844.94±661.79 | 603.23±481.46 | 1050.30±766.85 | 6841.88±10453.42 | 4355.48±4046.92 | 43.11±42.01 |

NA: not available.

# Table S9b. FM concentration in tissues of Ba/F3-*EPOR*-*JAK2*^V617F^ bearing mice after repeated oral administration (Mean ± SD; n = 6).

| Time-point (h) | Tissues (ng/g) | | | | | | | |
| --- | --- | --- | --- | --- | --- | --- | --- | --- |
|  | Brain | Heart | Liver | Lung | Kidney | Stomach | Intestine | Muscle |
| 0 | 6.30±3.68 | 7.06±1.49 | 12.95±3.13 | 18.51±4.00 | 16.62±1.41 | 71.78±44.34 | 21.35±19.10 | 18.63±11.72 |
| 0.5 | 282.56±93.75 | 1888.23±755.61 | 4356.13±3896.24 | 11417.84±1779.76 | 11797.01±3007.41 | 33064.98±22711.17 | 31165.68±12634.72 | 415.20±434.89 |
| 2 | 368.99±112.71 | 1324.53±674.49 | 6423.32±1255.84 | 7154.74±1884.31 | 7559.06±1994.60 | 19261.56±20678.63 | 4208.18±1319.95 | 274.79±157.58 |
| 4 | 492.40±753.97 | 577.67±355.57 | 6656.70±5814.19 | 3888.53±1467.55 | 4637.23±1862.84 | 11721.90±8596.23 | 9606.94±6016.15 | 181.26±120.06 |
| 8 | 47.02±31.27 | 357.37±375.51 | 1902.28±1325.16 | 1449.78±1432.68 | 1785.05±1445.33 | 3496.51±5193.87 | 1377.44±1368.99 | 84.95±73.10 |
| 12 | 10.82±4.65 | 15.80±11.28 | 150.74±116.18 | 132.40±76.67 | 173.59±120.65 | 2488.81±2532.45 | 249.53±337.40 | 13.27±6.38 |
