# Supplementary figures and images for: Preclinical studies of Flonoltinib Maleate, a novel JAK2/FLT3 inhibitor, in treatment of JAK2V617F-induced myeloproliferative neoplasms
Source: Blood Cancer J. 2022 Mar 7;12(3):37. doi: 10.1038/s41408-022-00628-2 (PMC8901636; doi:10.1038/s41408-022-00628-2)

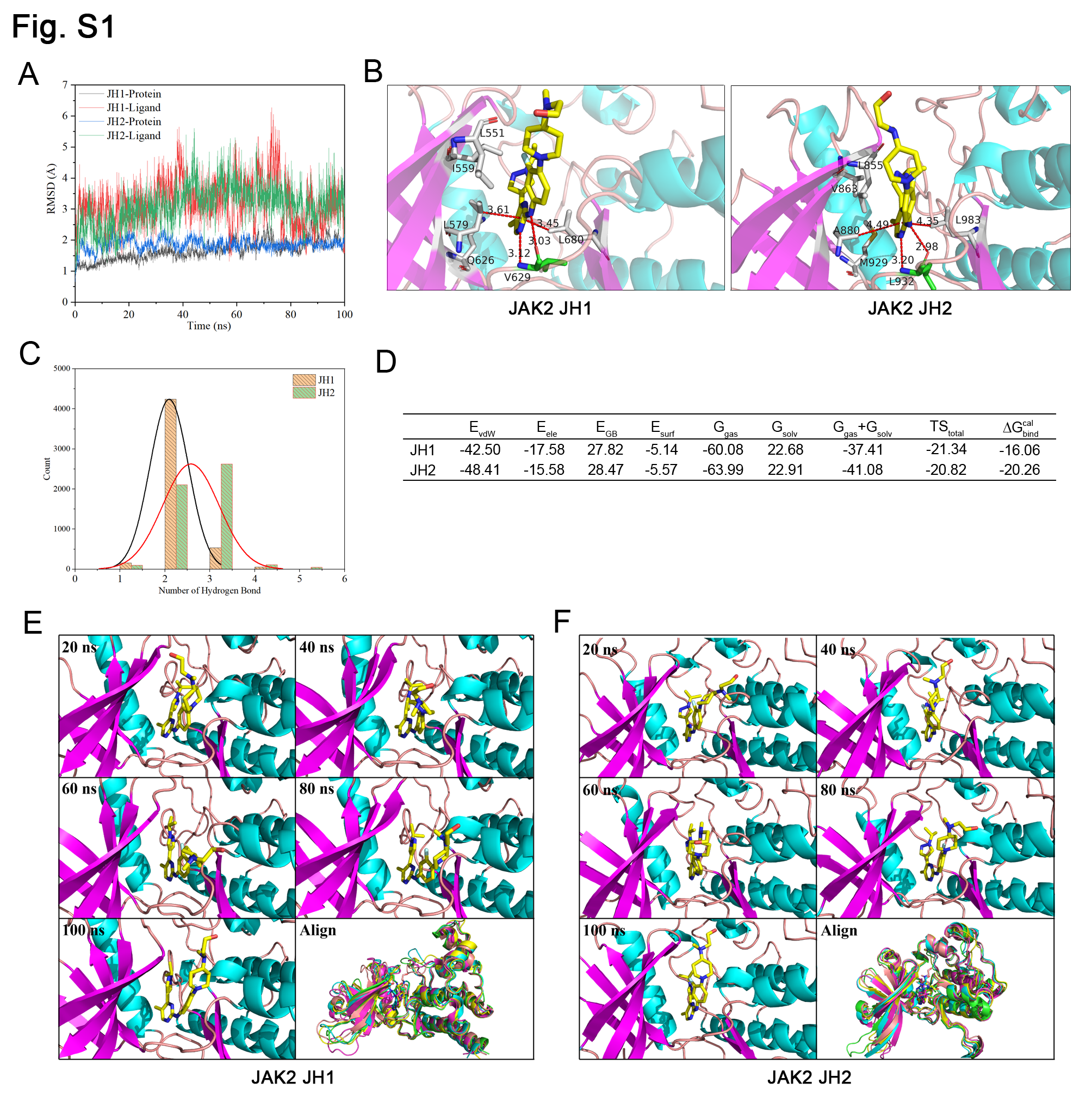

Supplement: Supplementary file 3 — Supplementary Figure 1 [file 41408_2022_628_MOESM3_ESM.tif]

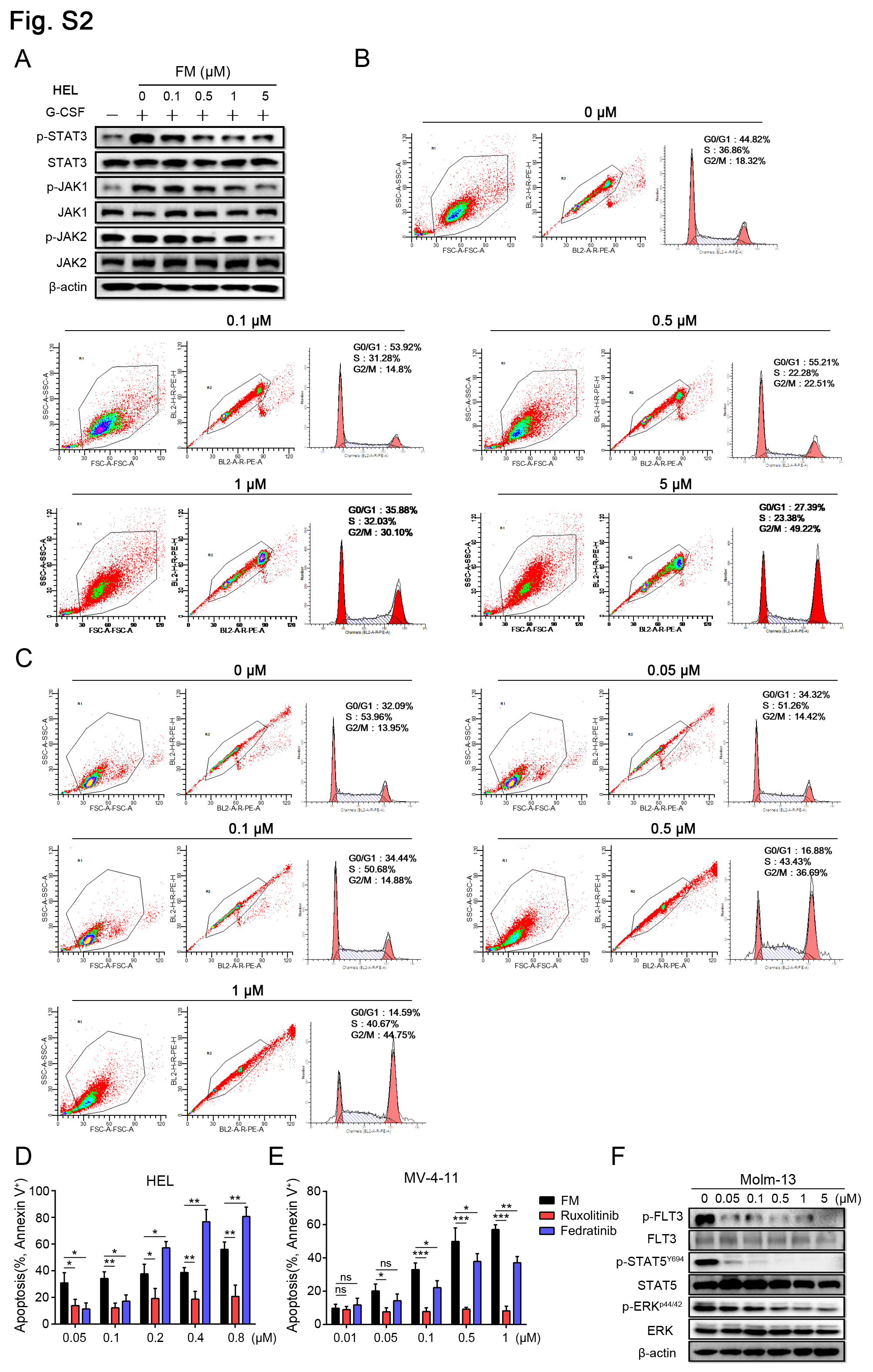

Supplement: Supplementary file 4 — Supplementary Figure 2 [file 41408_2022_628_MOESM4_ESM.tif]

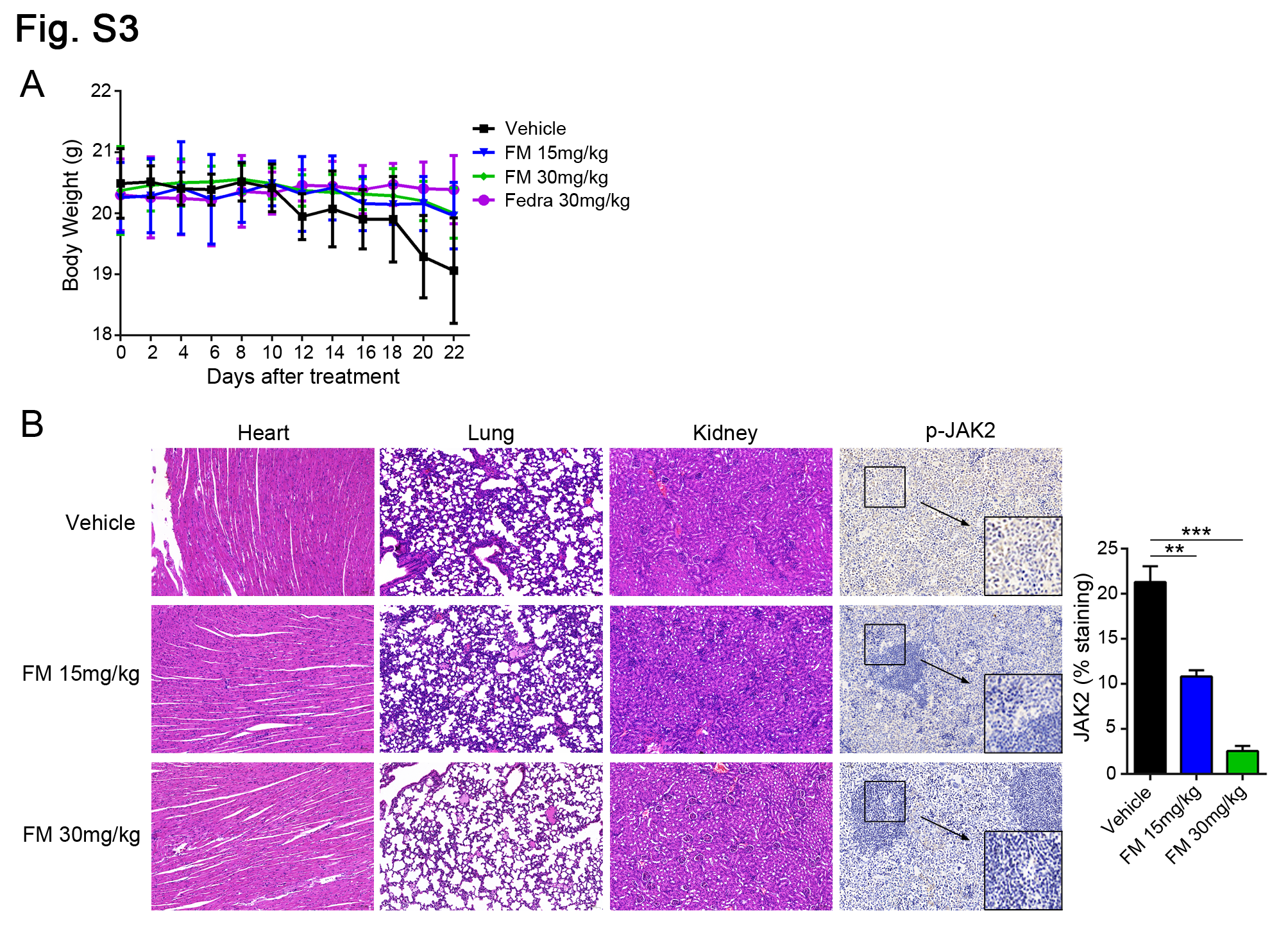

Supplement: Supplementary file 5 — Supplementary Figure 3 [file 41408_2022_628_MOESM5_ESM.tif]

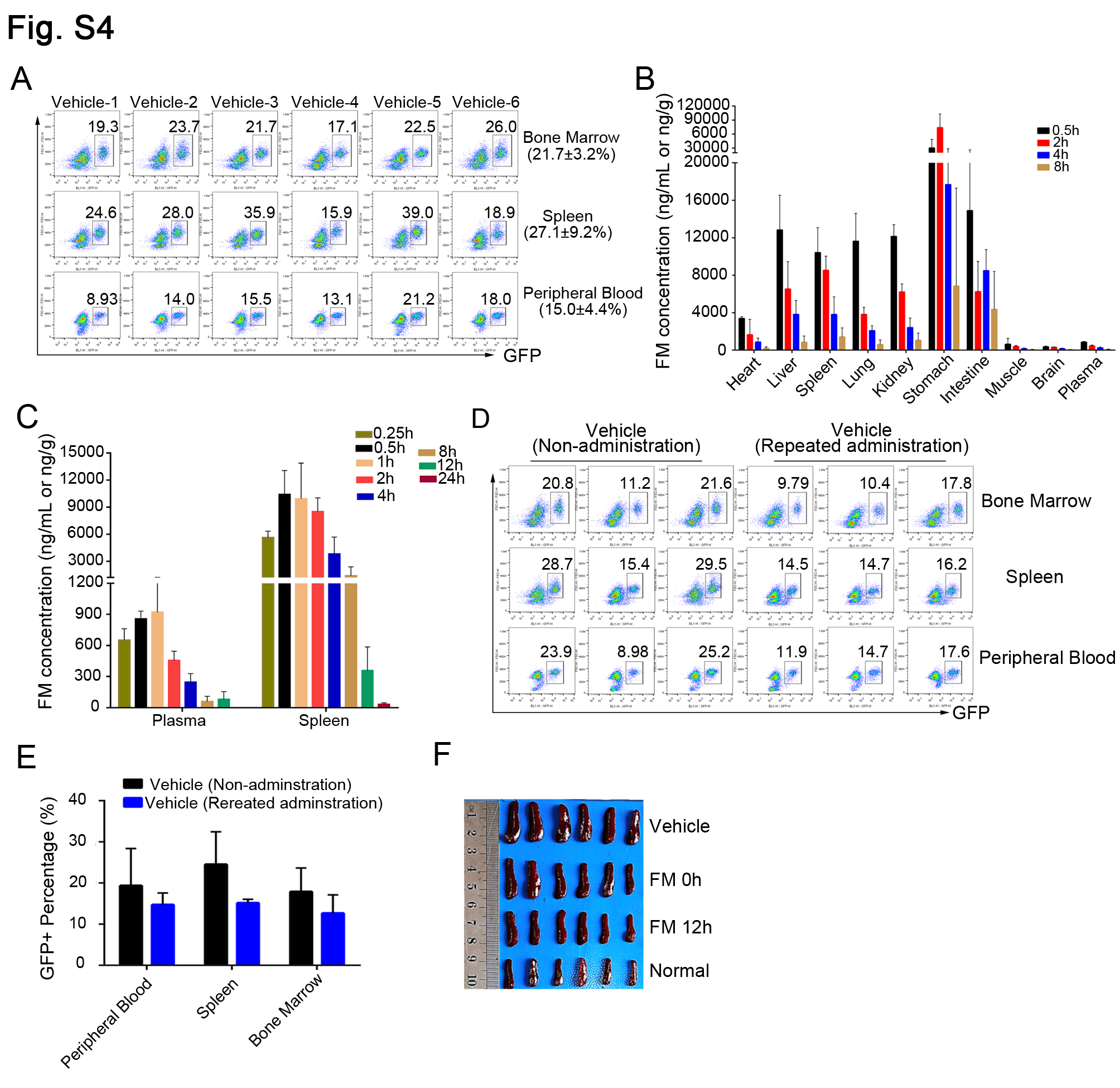

Supplement: Supplementary file 6 — Supplementary Figure 4 [file 41408_2022_628_MOESM6_ESM.tif]

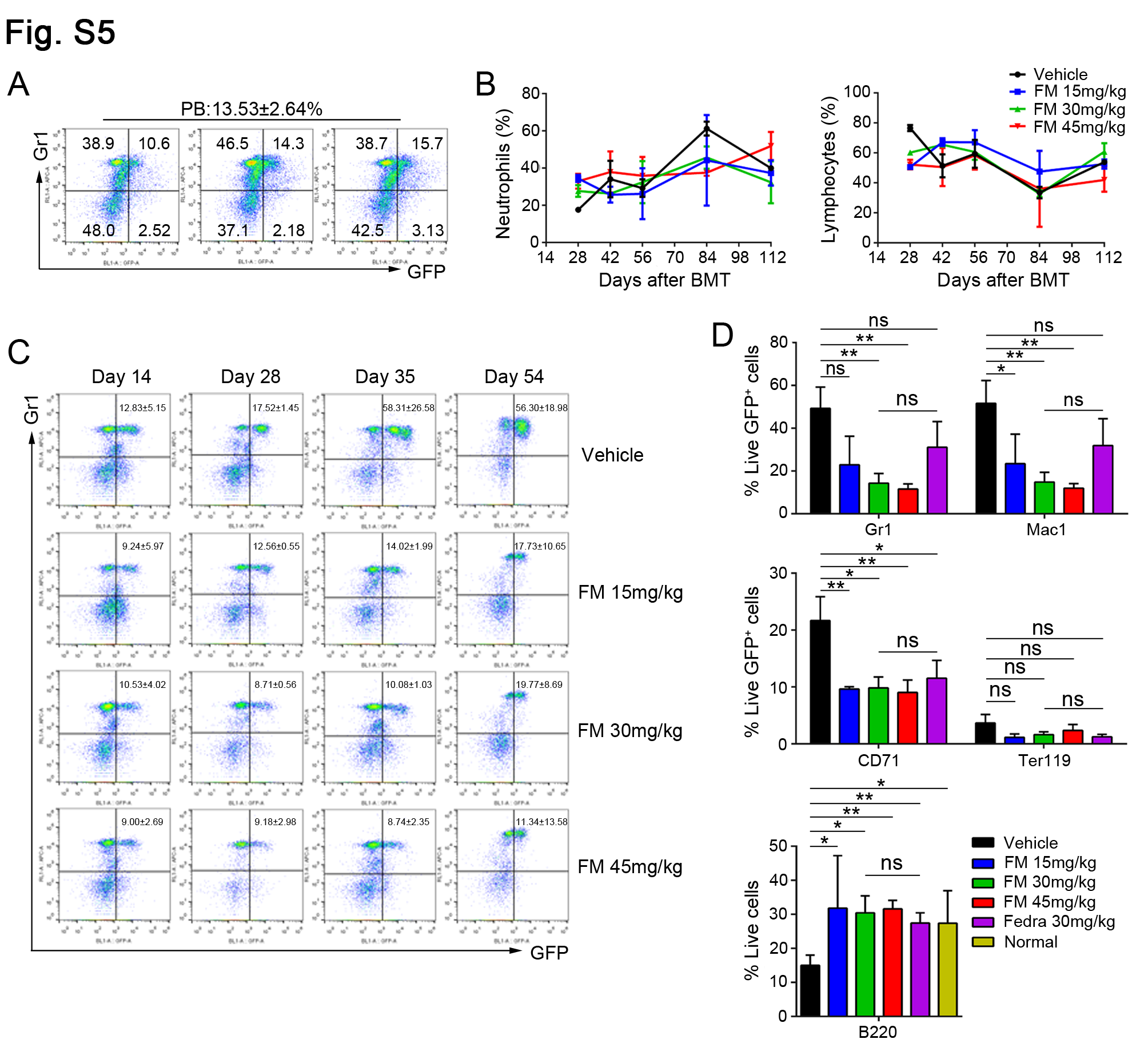

Supplement: Supplementary file 7 — Supplementary Figure 5 [file 41408_2022_628_MOESM7_ESM.tif]

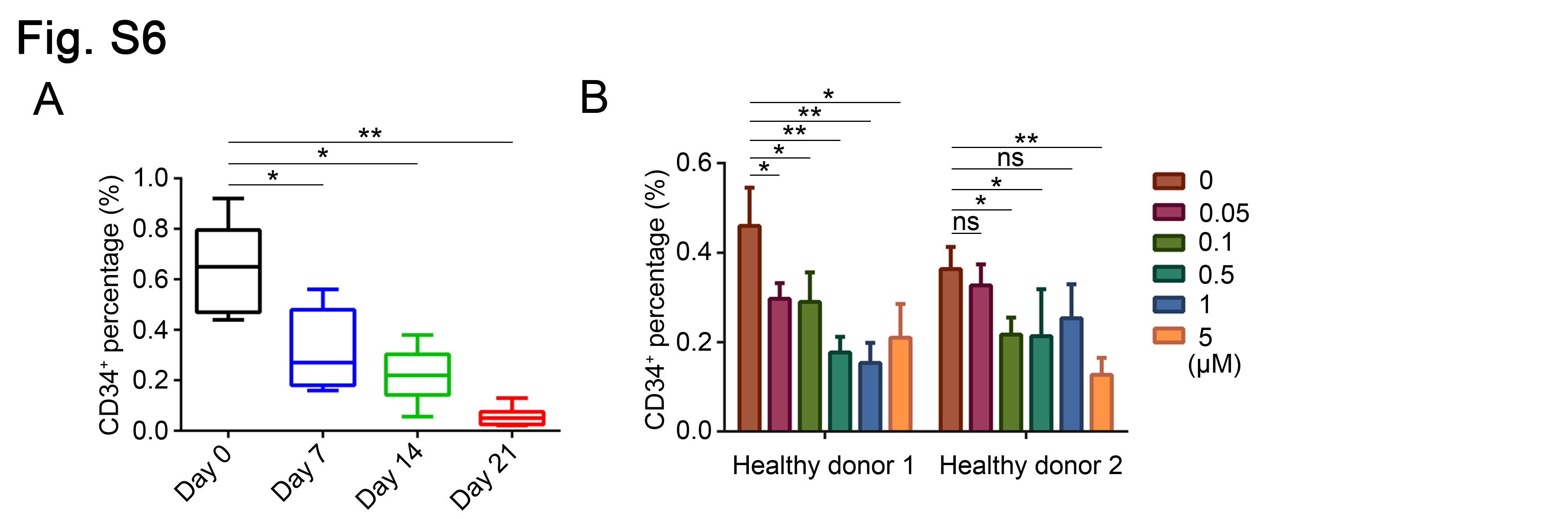

Supplement: Supplementary file 8 — Supplementary Figure 6 [file 41408_2022_628_MOESM8_ESM.tif]
